# *Pistacia lentiscus L*. revealed *in vitro* anti-proliferative activity on MCF-7 breast cancer cells and *in vivo* anti-mammary cancer effect on C57BL/6 mice through necrosis, anti-inflammatory and antioxidant enhancements

**DOI:** 10.1371/journal.pone.0301524

**Published:** 2024-04-18

**Authors:** Omayma Abidi, Zaineb Abdelkafi-Koubaa, Ilhem Bettaieb-Dridi, Lamjed Toumi, Lamjed Marzouki, Ouajdi Souilem

**Affiliations:** 1 Faculty of Sciences of Tunis, University Tunis El Manar, Tunis, Tunisia; 2 Laboratory of Physiology and Pharmacology, National School of Veterinary Medicine, University of Manouba, Sidi Thabet, Tunisia; 3 Laboratory of Personalized Medicine, Precision Medicine and Investigation in Oncology, Salah Azaiz Institute, University of Tunis El Manar, Tunis, Tunisia; 4 Laboratory of Biomolecules, Venoms and Theranostic Applications, Pasteur Institute of Tunis, University of Tunis El Manar, Tunis, Tunisia; 5 Immuno-Histopathology Service, Salah Azaiz Institut, Tunis, Tunisia; 6 Laboratory of Sylvo-Pastoral Resources, Sylvo-Pastoral Institute of Tabarka, University of Jendouba, Jendouba, Tunisia; 7 Unit of Functional Physiology and Bio-Resources Valorization (BF-VBR), Higher Institute of Biotechnology of Beja, University of Jendouba, Jendouba, Tunisia; 8 BiotechPole, Ariana, Tunisia; Sathyabama Institute of Science and Technology, INDIA

## Abstract

Inflammation and oxidative stress are two interconnected processes that play a role in cancer development and progression. In the present research, we aimed to evaluate the anticancer effect of *Pistacia lentiscus L*. (*PL*) essential oil (EO) *in vitro* against MCF-7 breast cancer cells and *in vivo* in DMBA-mammary cancer induction on female C57BL/6 mice model as well as to investigate its anti-inflammatory and antioxidant potential as implicated mechanism. Our results revealed a new chemotypes-profile of 39 bio-compounds of *PL* EO. The main chemotypes were terpenoid and ketone compounds. *In vitro*, *PL* EO had a potent anti-proliferative activity against MCF-7 cells. *In vivo*, *PL* reduced the tumor number, volume, weight and burden values as compared to the DMBA-positive control group (p<0.05). Histopathology data confirmed the protective effect of *PL* traduced by the presence of necrosis area. *PL* EO revealed improvement on inflammatory perturbation in the C-RP levels and the complete blood cell count. Finally, *PL* improved oxidative disorders of lipid peroxidation, thiol groups, hydrogen peroxide and antioxidant enzymes depletion in plasma and mammary tissues. Also, a potent plasma scavenging capacity has been detected. Our data suggested that *PL* chemotypes inhibited cell proliferation, exerting a potential protective effect against DMBA-mammary cancer through anti-inflammatory and antioxidant enhancements. Targeting inflammation and oxidative stress may represent a promising strategy for breast cancer prevention and treatment.

## Introduction

According to the World Health Organization (WHO), breast cancer (BC) is the most commonly diagnosed worldwide cancer in women with around 2,3 million women diagnosed in 2020 (24.2% newly diagnosed women/year). It is also the principal leading cause of women cancer-related death each year [1]. Every year 685,000 women die from BC. In Canada 78 Canadian women are diagnosed positive for BC [2]. In Africa, Polynesia has the highest mortality rate with around 85,800 women dying from BC in 2020 [1]. In Tunisia, it represents the first cause of mortality and morbidity from cancer (33%) with approximately 2,500 new cases every year [3]. Major risk factor associated with BC include age (risk increase with age), family history of BC (hereditary factor…), genetic mutation or other conditions such as alcohol consumption, smoking, prolonged exposure to sex hormone, exposure ionizing radiation at a young age, food additives and atmospheric pollutants such as polycyclic aromatic hydrocarbons (PAHs) [4, 5]. 7,12-dimethylbenz(a)anthracene (DMBA) belongs to PAHs, and it is a pure carcinogen substance that research work has proven the detection of mammary tumors in rodents, after the administration of DMBA [6, 7].

Also, inflammation and oxidative stress are two interconnected processes that play a role in cancer development and progression. It was proven that chronic inflammation such as gastric ulcer, hepatitis can contribute to the development of cancer like gastric and hepatic cancer development [8, 9].

Modern cancer treatment therapy focused on the use of a biological therapeutic approach based on immunotherapy, enzymotherapy, and derived from biologically natural substances such as: venom-proteins’ or plants’ bio-compounds [10–15]. *Pistacia lentiscus* L. (*PL*) (Anacardiaceae family) has been widely used in folk medicine in Mediterranean countries for millennia. In North Africa, leaves, drupes and mastic were traditionally used to treat circumcisions, diarrhea, eczema, icterus, nephrolithiasis, oral infections, respiratory wounds, skin burns and sore throats disorders [16]. It is also used in Tunisian ethno-pharmacy as antiseptic, cicatrizing and anticancer treatment [17]. This plant has a therapeutic potential against bacteria, fungi, viruses [18, 19], inflammation, oxidative damage, skin conditions and cancer [8, 20]. *PL* bio-compounds are used in cosmetics, in conservation of foods due to the important antimicrobial and antioxidant activities and as well as in pharmaceutical industries [21]. Several research works have proven that *PL* is characterized by important pharmacological properties against chronic diseases [20, 22] specially cancer such as human alveolar adenocarcinoma, neuroblastoma, leukaemia, thyroid carcinoma, sarcoma and colon cancer [23, 24]. No research study so far has explored the therapeutic effect of *PL* in the treatment of BC or mammary cancer (MC) in mouse models. As far as it could be ascertained, this is the first study investigating the *in vivo PL* EO protective effect against MC. Thus, the aim of the present study was to evaluate the anti-cancer potential of *PL in vitro* on MCF-7 hormone dependant breast cancer cells and *in vivo* DMBA-mammary cancer on C57BL/6 mice, to investigate the *in vivo* anti-inflammatory and antioxidant activities and to assess the *PL* extracts composition.

## Material and method

### Plant material

*PL* (Sapindales; Anacardiaceae) was harvested from the region of Tabarka, District of Jendouba, and Northeast Tunisia (Latitude: 36°57’16"N, Longitude: 8°45’29"E, altitude 108 m; annual rainfall 800–600 mm). Collected plants were identified and the certified specimen was deposited in the Herbarium of the National Research Institute of Rural Engineering Water and Forestry I.N.G.R.E.F-Tunisia under the reference VS1-PL2009. The landscape in Tabarka region is not polluted with absence of both domestic and industrial pollution. Samples were harvested during the male flowering season at the beginning of the month of March 2021 (red flower: 5 sepals from which 5 stamens emerge). EO extractions were immediately made by hydro-distillation following established protocols [25] and using distilled water as solvent. Encompassing leaves and flowers were delicately arranged within a receptacle. As the temperature reached its zenith at the boiling point (100°C), the aromatic essence from the plant akin to gasoline, which elegantly amassed on the container lid. Subsequently, the ethereal concoction underwent a metamorphosis within the refrigerant condenser, transition from a gaseous to a liquid state, entwining the essential oil and floral water in a seamless coil. The EO specimens were collected and stored at -20°C, until analysis.

### Cell lines

The human breast cancer cell lines estrogen receptor positive (MCF-7, ATCC^®^ HTB-22^™^)(*Homo sapiens*), were kindly provided by Pr. José Luis, Institute of Neurophysiopathology, Faculty of Pharmacy, University of Aix-Marseille, France.

### Chemicals reagents and media

Chemicals reagents were purchased from Sigma Chemical Co. (Sigma-Aldrich GmbH, SteinheimAlbuch, Germany): 7,12-dimethylbenz(a)anthracene, 1,1-Diphenyl-2 Picrylhydrazyl Radical, Gallic Acid, Quercetin, Catechin, Methanol, Sodium Chloride, Sulfuric Acid, Monosodium Phosphate, Sodium Hydroxide, Folin-Ciocalteu Reagent, Carbonic Acid Disodium Salt, Vanillin, Hydrogen Chloride, Aluminium Chloride, Cyclohexane, Methanol, Monosodium Phosphate, Hexaammonium Molybdate, Hydrogen Peroxide, Monopotassium Phosphate, Disodium Hydrogen Orthophosphate, Lauryl Sulphate Sodium, as well as Growth Media for the Culturing of Microbial Strains such as Mueller-Hinton Broth Agar, Luria-Bertani Agar Medium, Winge-Broth, Nutrient Agar, Agar, Trypto-Caseine-Soja, Sabouraud Agar, Mueller-Hinton Broth and Cancerous Cell Culture Reagents such as DMEM, Penicillin-Streptomycin Solution, EDTA, FBS, Trypsin, DMSO, Trypan Blue and Tetrazolium Salt MTT (3-(4,5-dimethylthiazol-2-yl)-2,5Diphenyl Tetrazolium Bromide).

### Animals

C57BL/6-female mice newly breastfed aged three weeks were purchased from the Pasteur Institute of Tunis. The handling of the animals was in the respect of the code of practice for the *Care and Use of Animals for Scientifïc Purposes* and the European Community guidelines (86/609/EEC) for animal-welfare. Mice were acclimated and housed in polypropylene cages under standard controlled conditions of the animal facility of the National School of Veterinary Medicine-Tunisia: 12/12 h light/dark cycle, 20 ± 2°C temperatures, 55% ± 15% humidity. Food and water were *ad-libitum*.

### Experimental design

This research adhered to the guidelines outlined in the Manual for the Proper Handling and Utilization of Laboratory Animals as per the National Institutes of Health’s directives [26–28]. The research plan obtained ethical clearance from the National School of Veterinary Medicine of Tunis Ethics Committee (Protocol ID Number: 14/2020/ENMV). All procedures are in compliance with directive 2010/63/EU of animal welfare (Articles 26, 30 and 33) and utmost care was taken to reduce any potential distress to the animals (Articles 4, 15, 17 and 39) [26]. The number of animals per group was reduced to 8 mice / group. Refinements were made to the protocol during the administration of DMBA, hormone and *PL* extract using gastric gavage to minimize pain and animal suffering. Animals were handled by the same person at the same time of the day: all actions were regrouped in harmonious successive actions to minimize stress (from 8 a.m. to 10 a.m.)[27, 28]. A planned experimental endpoint was predefined to anticipate severity and to stop the experimental procedure due to the harm loss and animal suffering using euthanasia method [29, 30]. All of the predefined conditions below can be reasons to stop experiment directly (in 2h and 24h at least) according to the article 15, 17 and 39 of the directive 2010/63/EU [26]: if we mentioned a body weight loss of 20% of the total body weight due to the decrease of food/water intake; if we checked important increase in tumor volume (diameter >1.5cm); if we checked respiratory disorders such as tachypnea or dyspnea after gavage; if we found an abnormal posture or lethargy / reluctance to move if stimulated or / and lack of movement; if we observed signs of suffering or anxiety such as isolation or withdrawn from other mice and expressed a ‘Pain face Grimace’ of semi-closed eyes and nose bulge; if we mentioned abnormal behavior like stereotypic movements and scratching the tumor until bleeding. Only 8% of animals were excluded and euthanized.

Thirty female mice were treated with DMBA at 20 mg/kg body weight, diluted in olive oil and administered per oral gavage using a stainless steel feeding needles for mices. Two weeks later, a second dose (50 mg/kg body weight) was injected in the left higher breast [31]. Animals received hormonal treatment with estrogen and progesterone for 5 weeks (Fig 2A). After DMBA administration animals were observed every 30 min for the first 4 h then every 1 h for 8 h and every 4 h for 24 h. Health status was checked for treated animals daily during 5 months using an Ethogram Template registration. Animals were deemed eligible for the study if they underwent a successful mammary cancer, which was defined as a mammary tumor diameter of 4 mm or more by measuring rule. Conversely, animals were excluded (16%) if they didn’t develop mammary cancer or if they presented any predefined sign of the experimental endpoint or if the animal experienced premature mortality, thereby preventing the acquisition of biochemical and histological data.

At the 19^th^ weeks, eligible mouse with mammary cancer were randomly distributed into three groups (n = 8) (Fig 2A):

- Group I (DMBA): animals served as DMBA positive control
- Group II (DMBA+ *PL*-1): DMBA mice received daily (5/2) *PL* EO (280 mg/kg b.w.)
- Group III (DMBA+ *PL*-2): DMBA mice received daily (5/2) *PL* EO (570 mg/kg b.w.)

Animals Treatment with *PL* EO was carried out for 4 weeks in the same conditions. Until the end of 5 months of the whole process, mice of three groups were euthanized in the same date by decapitation according to the *American Veterinary Medicine Association Guidelines for the Euthanasia of Animals*[32].

### Institutional review board statement

The animal study protocol was approved by the Ethics Committee of the NATIONAL SCHOOL OF VETERINARY MEDICINE Sidi Thabet-Tunisia (CEEA-ENMV): Approval ID code: 14/2020/ENMV, Approval date: 30.03.2020 Sidi Thabet-Tunisia.

### Gas chromatography-mass spectrometry analysis (GC-MS)

Chemometric profiling was performed using a GC-MS system (Thermo FïsherScientifïc, Walthan, Massachusetts, USA). The extracts were solubilised in methanol (1% v/v) and 1 μL of each sample was injected in a split mode (ratio 15: 1) for 75 min, using Agilent GC7890B gas chromatography instrument coupled with an Agilent MS 240 Ion Trap (Agilent, CA, USA). The separation was accomplished in a HP-5MS capillary column (30 m×250 μm, fïlm thickness: 0.25 μm). Helium (99.99%) was used as carrier gas, released at a constant flow rate of 1 mL/min. The initial oven temperature started at 40°C, maintained for 2 min, then increased 5°C/min to 250°C, and held constant at this temperature for 20 min. The injector temperature was set at 280°C. The detection was made in full scan mode for 60 min. Mass spectrometry (MS) operating parameters were as follows: ion source temperature: 200°C, interface temperature: 280°C, ionizing electron energy (EI) mode: 70 eV, scan range: 50–1,000 m/z. Bio-compounds interpretation and identification were performed by comparing mass spectra with those referenced in the NIST 05 database (NIST Mass Spectral Database, PC-Version 5.0, 2005 National Institute of Standardization and Technology, Gaithersburg, MD, USA).

### *In vitro* anti-cancer activity

After thawing, MCF-7 cells were trypsinized and placed in Dulbecco’s modified eagle medium (DMEM) in new flasks, supplemented with 10% foetal bovine serum (FBS) and 1% penicillin/streptomycin. Cells were maintained in 5% CO_2_ humidified atmosphere at 37°C. The growth medium was changed when 80% of the cells where confluent (approximately three times a week) for 2–3 weeks. Cell culture was maintained until obtaining a concentration of 10^5^ cells/mL [33].

MCF-7 cell line was seeded in 96-well culture plates at appropriate concentration (10^4^ cells/ well). After an overnight incubation, the medium was changed and cells were incubated with either medium alone or with increased concentrations of *PL* extract. After either 24h or 72h treatment incubation, cellular viability and proliferation were assessed using the 3-(4,5-dimethlthiazol-2-yl)-2,5-diphenyltetrazolium bromide tetrazolium reduction colorimetric MTT assay [33]. Cells were lysed by DMSO and absorbance was measured with a microplate reader (Multiskan, Lab systems, GmbH) at 560 nm wavelengths. The untreated cells, containing only medium, were used as control. The results were expressed as percentages of viable cells compared to non-treated cells taken as controls.

### *In vivo* anti-cancer potential

#### Measurement of body weight, tumor volume and burden

Weight gain was taken regularly each week during our study, from the start of the experiment until euthanasia. Tumor weight and yield means were the mean tumor weight and number per group taken after euthanasia. The tumor volume defined using this formula: v = (π/6)×(length × width^2^). The tumor burden is determined by multiplying Tumor volume by the number of tumors per mouse.

Weekly measurement of the mammary cancer sizes >1 mm in diameter was carried out using calipers.

#### Histopathological analysis

Directly after euthanasia, mammary cancer tissues were removed, washed with NaCl (0.9%) and were preserved in a buffered neutral formalin solution (10%). After dehydration with ethanol then xylene, samples were finished in paraffin to be cut into 2μm thick sections. Putting on the slides, the sections were deparaffinized, hydrated (with decreasing concentrations of ethanol) to facilitate their staining with hematoxylin and eosin.

### Anti-inflammatory activity

The complete blood cell count test (CBCC) and C-reactive protein (C-RP) content were carried out in the biochemical laboratory, hospital Rabta-Tunisia. C-RP was assessed using diagnostic kits (BioSystems S.A., Costa Brava 30, 08030 Barcelona, Spain, certifïed according to ISO 13485 and ISO 9001).

### Antioxidant effect

#### Oxidative stress biomarkers

Mammary and plasmas lipid peroxidation (MDA) was detected by thiobarbituric acid reactive (TBARS) content according to standard method [34]. The MDA levels were performed by using an extinction coeffïcient for the MDA-TBA complex of 1.56 1.M^− 1^ cm^− 1^ and expressed as nmol/mg protein in mammary tissues and nmol/mL in plasma. Thiol group concentration (-SH) was determined according to a standard method [35]. Results were expressed as nmol /mg of protein in mammary tissues and nmol/mL in plasma. Hydrogen peroxide was measured and expressed in nmol/mg protein in mammary tissues and nmol/mL in plasma [36].

#### Antioxidant enzyme stress biomarkers

The determination of enzymatic antioxidant activities was accomplished by detection of the superoxide dismutase enzyme SOD activity [37] and expressed as Unity U SOD/mg of protein in mammary tissues and UI SOD /mL in plasma. The catalase activity CAT by measuring the initial rate of H_2_O_2_ disappearance [37] and expressed as μmol H_2_O_2_ /min/mg of protein in mammary tissues and μmol H_2_O_2_ /min/mL in plasma. Peroxidase activity was performed according to standard method [38] and expressed as UI/mg of protein in mammary tissues and UI/mL in plasma.

#### Plasma scavenging capacity

Plasma in the three groups was subjected to 2,2-diphenyl-1-picryl-hydrazyl-hydrate (DPPH) free radical scavenging method with slide modifïcations [39]. Briefly, at room temperature, 100 μL of different plasma’ groups were mixed with 2 mL of DPPH freshly prepared methanolic solution (0.1 mM). The samples were stored in the dark, at room temperature then centrifuged. The DPPH radical reduction was estimated at 516 nm wavelength. The inhibition (%) of the DPPH free radical scavenging ability was measured as a function of plasma concentrations as follows:

$$\text{Plasma}\;\text{scavenging}\;\text{capacity}\;\left(\%\right)=100\text{x}\;\left[\left({\text{Abs}}_{\text{control}}-{\text{Abs}}_{\text{sample}}\right)/{\text{Abs}}_{\text{control}}\right]$$

Where: Abs _control_ is absorbance of pure DPPH and Abs _sample_ is absorbance of plasma samples.

### Statistical analysis

Data was analysed using GraphPad Prism 8.4.2 Software (La Jolla, CA, USA). Data was expressed as the mean ± standard error (SE). The statistical difference between different groups was tested using the Kruskal-Wallis non-parametric test. The difference was considered signifïcant at the 5% threshold (*p*< 0.05). All experiments were carried in triplicate.

## Results

### Phytochemical composition

The GC-MS chromatographic analysis of *PL* EO showed 54 peaks detected in 99.32% of the extract (S1 Fig in S1 File). Peaks corresponded to 39 bioactive compounds (Table 1 and S1 Fig in S1 File), recognized relating to their retention time (min), peak area (%) and mass spectral fragmentation patterns compared to compounds of the NIST library. The main chemotypes were: (1R) 2,6,6-Trimethylbicyclo (3.1.1)hept-2-en-4-one (1R-verbenone) (26.6%); 1-methylene-4-(1) (23.6%); D-limonene (13.2%); 4-Terpinenyl acetate (7.7%); 3,7-dymetyl-1,3,6-Octarienne (6.09%); alpha,-Phellandrene (5.4%). *Pistacia lentiscus* EO contained low concentrations (< 2%) of Caryophyllene; Trans-.beta.-Ocimene; Santolinatriene; Humulene; Germacrene D; alpha.-Farnesene;… Compounds were ranked according to their elution on Rtx-5MS capillary column in (Table 1 and S1 Fig in S1 File).

**Table 1:** Phytochemical volatile compounds composition in *Pistacia lentiscus* essential oil using gas chromatography-mass spectrometry.

Peaks N°	RT (min)	Library identified compound name /ID	Area (%)	Chemical class	Molecular formula
1	10.361 13.602	3-Carene	0.413	Monoterpene	C_10_H_16_
2	10.631	Bicyclo(3.1.0)hex-2-ene, 2-methyl-5-(1-methylethenyl)-	0.412	Ketone	C_10_H_14_
3	10.917	(1R)-2,6,6-Trimethylbicyclo (3.1.1)heptane	26.627	Ketone	C_10_H_18_
4	11.357	Santolinatriene	1.212	Organichydrocarbon	C_10_H_16_
5	12.454 13.012 13.866 16.271	1-methylene-4-(1-methylcyclo propyl)-1-cyclohexene	23.671	Ketone	C_11_H_18_
6	13.424	.alpha.-Phellandrene	5.441	Cyclicmonoterpene	C_10_H_16_
7	14.141	Tert-Butylbenzene	2.918	Benzenederivative	C_10_H_14_
8	14.328	D-Limonene	13.211	Monoterpene	C_10_H_16_
9	14.646	Trans-.beta.-Ocimene	1.932	Monoterpene	C_10_H_16_
10	15.022	3,7-dymetyl-1,3,6-Octarienne	6.091	Plant metabolite	C_10_H_16_
11	15.347	4-Terpinenyl acetate	7.776	Monoterpenic ester	C_12_H_20_O_2_
12	15.022	1,6-Octadien-3-ol, 3,7-dimethyl-Linalool	0.103	Cyclicmonoterpene	C_10_H_18_O
13	16.846	3-methyl-, 3-m-tolyl- Butanoic acid, 3-methyl-2-oxobutanoic acid	0.220	Fattyacidmethyl ester	C_12_H_16_O_2_
14	17.179	(E)-4,8-Dimethylnona-1,3,7-triene 2,6-dimethyl 2,6,8nonatriene	0.159	Acyclichydrocarbonalkatriene	C_11_H_18_
15	17.564	2,4,6-Octatriene, 2,6-dimethy	0.158	Ocimene	C_10_H_16_
16	19.087	Terpinen-4-ol	2.412	Monoterpene	C_10_H_18_O
17	19.323	11-(2-Cyclopenten-1yl)undecanoicacid	0.112	Acid hydnocarpicacid	C_16_H_28_O_2_
18	22.385	2-Pentacosanone	0.534	Ketone	C_25_H_50_O
19	19.452	.alpha.-Terpineol	0.374	Alcool monoterpene	C_10_H_18_O
20	20.784	Acetic acid, trifluoro-, nonyl ester	0.028	Monocarboxylic acid	C_11_H_19_F_3_O_2_
21	22.188	ABornyl acetate	0.405	Monoterpene	C_12_H_20_O_2_
22	21.155	Isopentyl hexanoat	0.194	Fattyacid ester	C_11_H_22_O_2_
23	23.903	Camphene	0.121	Bicyclicmonoterpene	C_10_H_16_
24	24.636	.alpha.-copaene	0.101	Sesquiterpene	C_15_H_24_
25	25.790	Caryophyllene	0.701	Sesquiterpene	C_15_H_24_
26	26.162	1-Butanol, 3-methyl-, benzoat	0.213	Ester monoterpene	C_12_H_16_O_2_
27	26.545 27.198 31.104 31.373	.gamma.-Muurolene	0.292	Sesquiterpene	C_15_H_24_
28	26.642	Humulene	0.302	Sesquiterpene	C_15_H_24_
29	26.642	(-)-Aristolene	0.139	Bicyclicsesquiterpene	C_15_H_24_
30	27.125 28.338	1-Isopropyl-4,7-dimethyl-1,2,delta-Amorphene	0.881	Sesquiterpene	C_15_H_24_
31	27.328 27.591	Germacrene D	0.380	Sesquiterpene	C_15_H_24_
32	27.922	.alpha.-Muurolene	0.376	Sesquiterpene	C_15_H_24_
33	27.922	.alpha.-Farnesene	0.972	Sesquiterpene	C_15_H_24_
34	28.112	Naphthalene	0.065	benzenoid polycyclic aromatic hydrocarbon	C_10_H_8_
35	28.553	Cubenene	0.072	Sesquiterpene	C_8_H_4_
36	29.411	3-Hexen-1-ol,benzoate,(Z)-	0.149	Ester of benzoic acid	C_13_H_16_O_2_
37	30.789	.alfa.-Copaene	0.064	Sesquiterpene	C_15_H_25_
38	31.986	(1R,4R,5S)-1,8-Dimethyl-4-(prop-1-en-2-yl)spirodec-7-ene	0.033	Sesquiterpene	C_15_H_24_
39	33.730	Benzyl Benzoate	0.056	Monoterpenic ester	C_14_H_12_O_2_
		Monoterpenes	24.412		
		Sesquiterpenes	04.313		
		Hydrocarbons	01.436		
		Ester	08.608		
		Ketone	51.244		
		Others	09.307		
		Total identified	99.320		

Compounds were listed in order of their elution on Rtx-5MS capillary column. Interpretation and identification were based on a comparison of the compounds mass spectral data and Koyats retention indices (RI) with those of NIST Mass Spectral 05 Library database (2011), Wiley Registry of Mass Spectral Data 8th edition and literature. The data presented were the mean values of three replicates.

### *In vitro* anti-cancer activity

**Fig 1:**
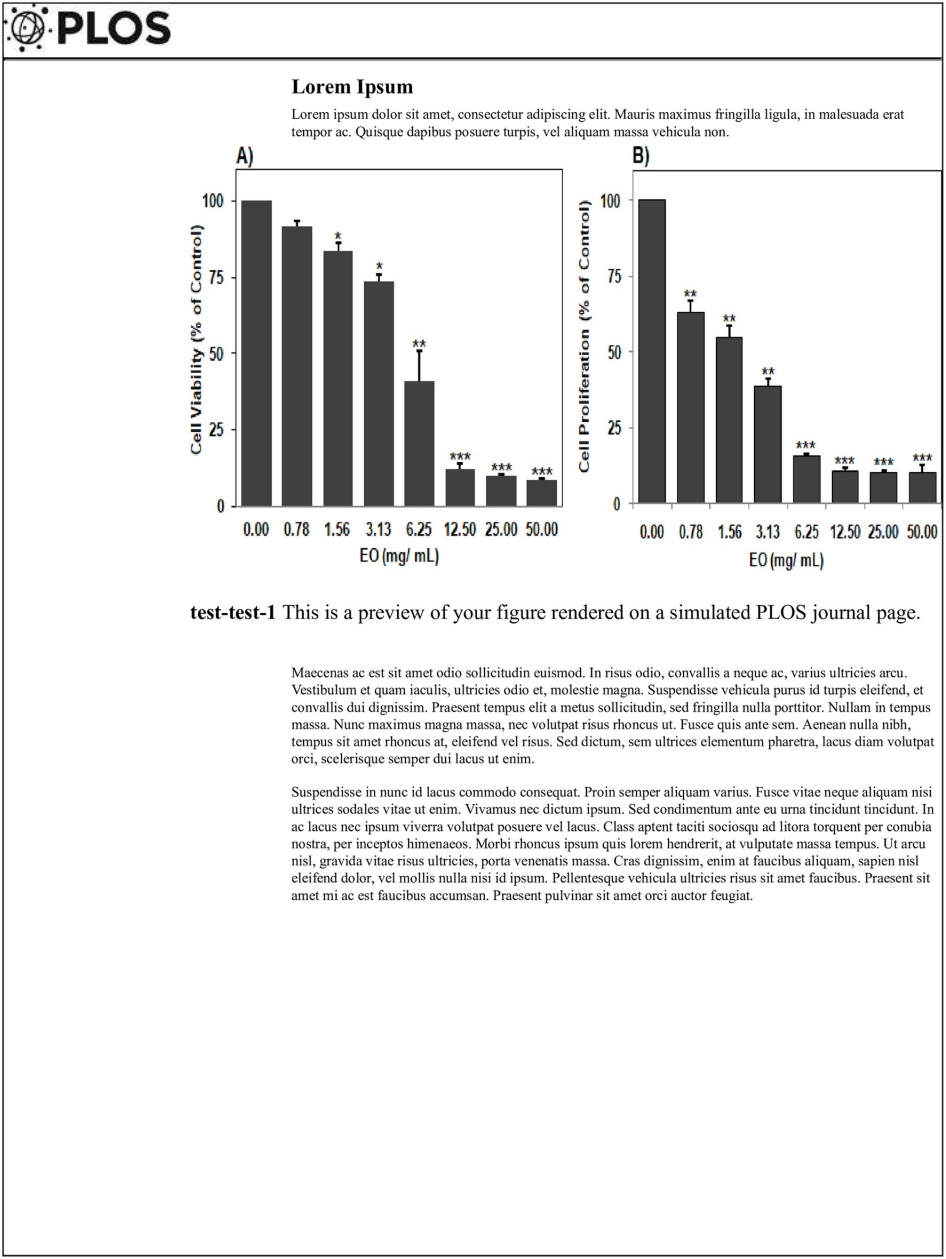
Effect of essential oil of *Pistacia lentiscus* on MCF-7 cell viability (1A) and cell proliferation (1B). Cells were treated with increasing doses of *PL* EO extract for 24h and 72h. Cell viability was estimated by metabolic rate using the MTT assay. Absorbance values were measured at 560 nm wavelength and compared to untreated cells (containing medium alone). Data represent the mean ± standard error of mean (SEM) of two independent experiments performed in triplicates.

MTT assays showed that *PL* EO had a significant effect on MCF-7 cell viability after 24 h treatment with 0–50 mg/ mL (Fig 1A). Furthermore, *PL* EO displayed potent anti-proliferative activity against MCF-7 after 72 h treatment (Fig 1B). Interestingly, the anti-proliferative activity was in a dose-dependent manner with IC_50_ value of 2 mg/mL.

### *In vivo* anti-cancer potential

#### Measurement of body weight, tumor volume and burden

**Fig 2:**
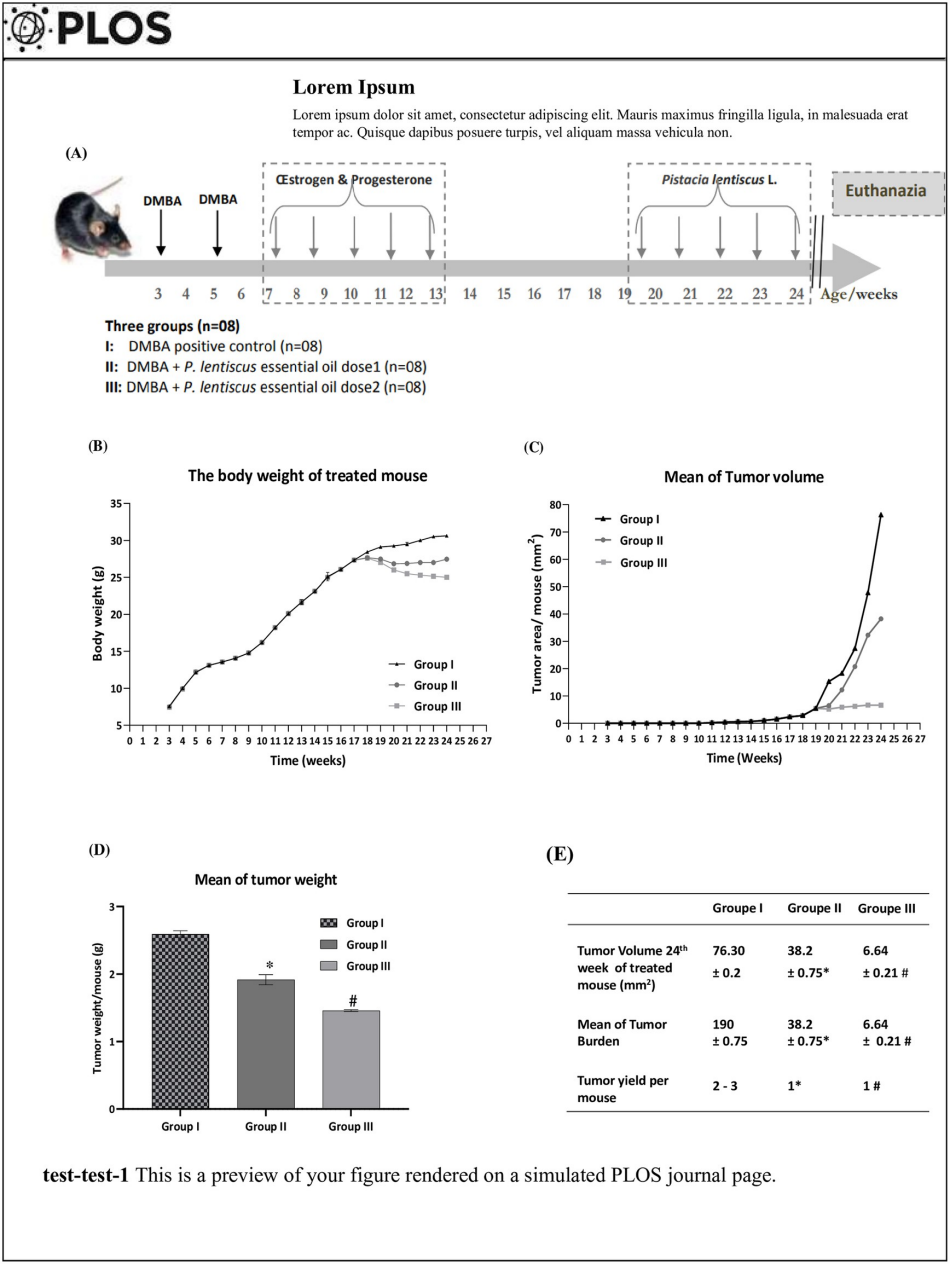
*Pistacia lentiscus* essential oil protective effect on DMBA-mammary cancer in C57Bl/6 female mice. Twenty four C57Bl/6-female mice were treated as described in the experimental design of Section Material and Method: All animals were pre-treated with two doses of DMBA (20 and 50mg/kg, b.w. dose) associated with progesterone and estrogens treatments until mammary tumor induction. GroupI: had not received any treatment after DMBA-mammary cancer induction, while group II and III received *PL* EO (280 and 570 mg kg, b.w. dose. respectively) after DMBA mammary cancer induction. (2A). Measurement of body weight gain (2B) and the volume of mammary tumours growth (2C) were measured during the whole process for 5 months as described in Measurement of body weight, tumor volume and burden of Section Material and method. The tumours’ weight per mouse in each group (2D) was measured after euthanasia. The average numbers of mammary tumours in each mouse and the mean of tumour burden (2E) were calculated in various tumour diameter groups at the end of the process. Values are given as median and (minimum value- maximum value), *p<0.001: Compare to group I, positive control DMBA (ANOVA test) and # *p*<0.001: Compare to group II (ANOVA test).

To explore the effect of *PL* EO, the initiation of mammary carcinogenesis in female C57BL/6 mice was induced by DMBA and promoted by hormone treatment (Fig 2A). Firstly, body weight measurement was performed throughout the treatment period. No significant difference was marked between the three groups until the 15^th^ week of the experiment (18^th^ week of age). However, the body weight was significantly reduced in the co-treated groups with *PL*. In addition, the reduction in body weight is dependent on the PL dose. In fact, the reduction of weight in the group III (treated with *PL* 2) was more signifïcant (Fig 2B).

Secondly, the tumor mass began to appear during the 9th week of treatment (12^th^ week of age). Tumor growth was consistently elevated and equivalent in the three groups in terms of size and volume. Up to the 15^th^ week of treatment, the tumor volume of the positive control group increased linearly from 2.36 mm^2^ (16^th^ week of treatment) to 76.21 mm^2^ at the end of the study. The co-treatment with *PL* significantly reduced volume from the beginning of co-treatment to the end of the study with values of 38.2 and 6.64 in the group *PL*-1 and *PL*-2, respectively (Fig 2C). Thirdly, the increase in tumor volume was associated with the increase in weight especially in the DMBA positive control group (2.5g). The weight of the tumors was reduced after the administration of EO and reached 1.9 and 1.4 g in the co-treated groups *PL*-1 and *PL*-2, respectively (Fig 2D). Finally, the exposure to DMBA triggered a multiplicity of the number of mammary tumors in the positive control group and thus increasing tumor volume and burden which were reduced in the co-treated mice, presenting a reduced number of tumors yield per mouse, a smaller volume tumor mass and an equally reduced tumor burden value (Fig 2E) compared to positive control group (*p*<0.05).

#### Histopathological analysis

**Fig 3:**
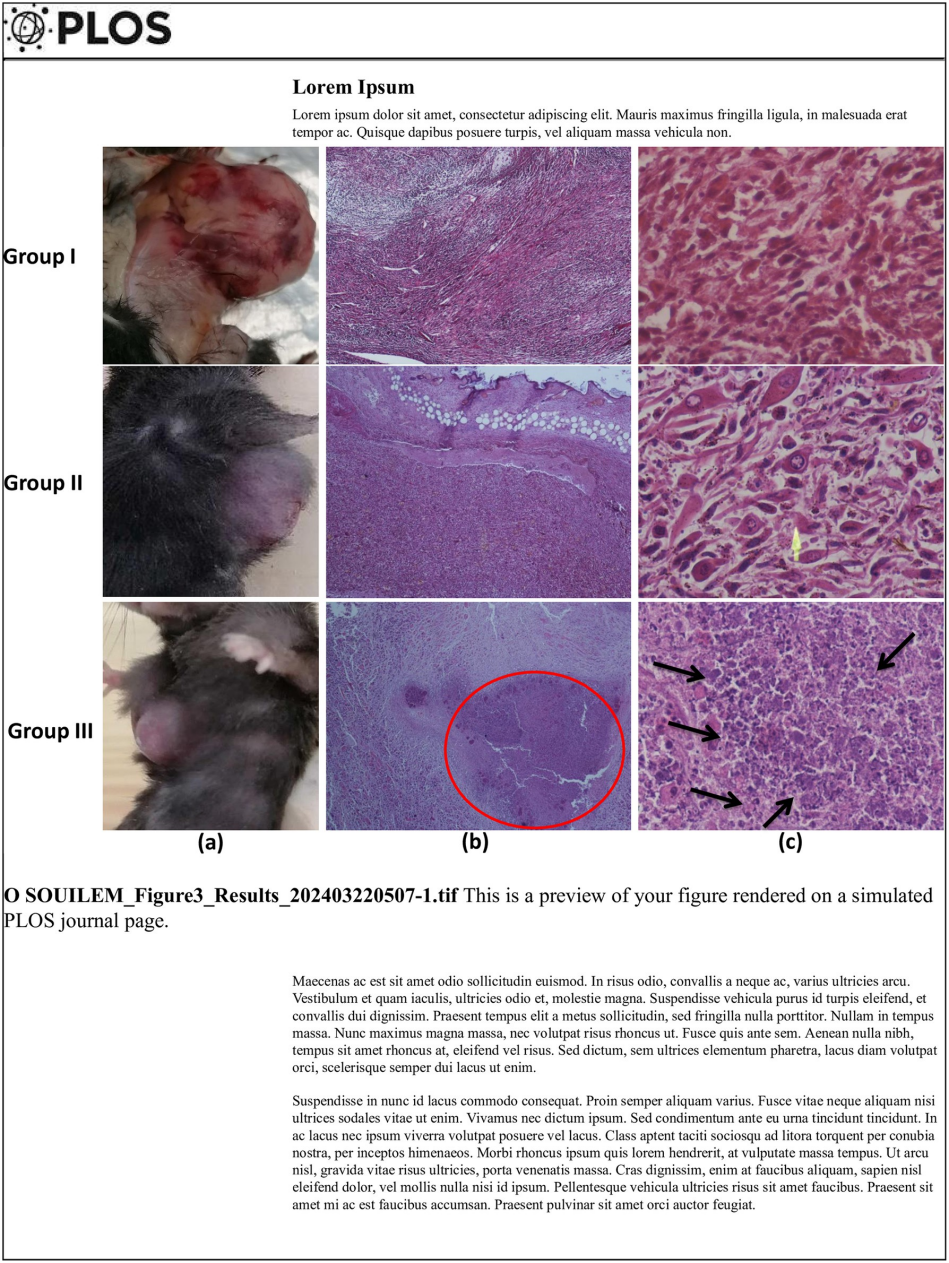
Protective effect of *Pistacia lentiscus* essential oil on carcinogenesis process induced mammary cancer in female C57Bl/6 mice. Microscopic observation (Real picture on the left, X10 on the middle, X40 on the right) in the mammary tissues of group I: DMBA positive control, Group II: DMBA then *PL*-1(280 mg/kg-b.w. dose) (CTR^+^ +*PL*-1) and Group III: DMBA then *PL*-2 (560 mg/kg-b.w. dose) (CTR^+^ + *PL*-2). All animals were pre-treated with two doses of DMBA (20 and 50mg/kg, b.w. dose) associated with progesterone and estrogens treatments until mammary tumor induction. GroupI: had not received any treatment after DMBA-mammary cancer induction, while group II and III received *PL* EO (280 and 570 mg/ kg, b.w. dose. respectively) after DMBA mammary cancer induction. Red circle and black arrow: necrosis areas; yellow arrow: atypical and pleomorphic mammary cells.

To determine *PL* protective effect, we microscopically investigated the hematoxylin and eosin (H&E) stained DMBA-mammary cancer tissues from female C57BL/6 mice from the three groups (Fig 3). The macroscopic examination in group I (DMBA) indicated that mammary cancer was solid and bulky (volume = 76.21 mm^2^ while the histological examination indicated a disorganized, atypical and pleomorphic mammary cells, indicating an invasive malignant tumor: sarcoma. In contrast, treatment with *PL* EO in group II reduced DMBA-mammary tumor growth in the used *PL*-1 group (volume = 38.2 mm^2^). The histology dissection illustrates change in mammary tissues that infiltrated the dermis cells: infiltrating fat phenotype with a pleomorphic epithelioid appearance. Moreover, an important reduction of DMBA mammary cancer was accomplished in the group III (*PL*-2) (volume = 6.64 mm^2^). On histological dissections, we observed sarcomatous tumors with necrosis area and the presence of a few mammary glands.

### Anti-inflammatory activity

Complete Blood Cell Count Analysis and serum C-RP activity (Table 2) were significantly increased in the positive control group (C-RP: 1.96μg/dL; WBC: 17.5 (10^9^)/L; Mid: 0.4 (10^9^)/L) when compared to *PL* co-treated groups (1 and 2) (*p*<0.05). Interestingly, we showed a corrective effect of *PL*-1 *EO* against the chronic inflammation caused by DMBA-mammary cancer in the C-RP activity and CBCC levels (C-RP: 1.2μg/dL; WBC: 15.9 (10^9^)/L; Mid: 0.29 (10^9^)/L). More importantly, we found a powerful anti-inflammatory activity in the group PL-2 when compared to the group *PL*-1 (*p*<0.05) in the C-RP activity and CBCC levels (C-RP: 1 μg/dL; WBC: 15.1(10^9^)/L; Mid: 0.2 (10^9^)/L) which prove the efficacy of *PL*-2 dose more than *PL*-1 (Table 2).

**Table 2:** Evaluation of *Pistacia lentiscus* essential oil anti-inflammatory potential on DMBA mammary cancer model using hematological parameters of complete blood cell count and C-RP levels.

CBCC/C-RP	Blood sample ± SE		
	G I	G II	GIII
**WBC (10** ^**9**^ **/L)**	**17.5± 0.21**	**15.9± 0.12** *	**15.1± 0.23** ^#^
Lymph (10^9^/L)	13.04± 0.08	12.9± 0.15 *	12.8± 0.1 ^#^
**Mid (10** ^**9**^ **/L)**	**0.4± 0.21**	**0.29± 0.32** *	**0.2± 0.23** ^#^
Gran (10^9^/L)	1.89± 0.5	1.8± 0.12 *	1.78± 0.14 ^#^
HGB (g/dL)	9.3± 0.22	10± 0.45 *	10.3± 0.36 ^#^
RBC (10^12^/L)	7.19± 0.24	8.9± 0.12 *	9.1± 0.13 ^#^
Htc (l/l)	0.37± 0.05	0.48± 0.07 *	0.5± 0.09 ^#^
MCV (fL)	45.2± 0.11	49.5± 0.23 *	52.1± 0.32 ^#^
MCH (pg)	14± 0.5	14.6± 0.3 *	14.9± 0.4 ^#^
MCHC (g/dL)	24.2± 0.12	25.1± 0.2 *	25.8± 0.09 ^#^
**PLT (10** ^**9**^ **/L)**	**1103± 5**	**978± 7.5** *	**980± 2.3** ^#^
**C-RP (μg/dL)**	**1.96 ± 0.37**	**1.2± 0.21** *	**1± 0.37**

All animals were pre-treated with two doses of DMBA (20 and 50 mg/kg, b.w. dose) associated with progesterone and estrogen treatment until mammary tumor induction. Group I: had not received any treatment, while group II and III received essential oil of *P*. *lentiscus* (280 and 570 mg/kg, b.w. dose, respectively). Results are represented by the mean ± standard error (SE) (n = 08).

*: *p* < 0.05 vs Group I, and

^#^: *p* < 0.05 vs Group II.

CBCC: Complete Blood Cell Count; WBC: White Blood Cells count; Lymph: Lymphocytes; Mid: Monocytes; Gran: Granulocytes; HGB: Hemoglobin; RBC: Red Blood Cells count; Htc: Hematocrit; MCH: Mean corpuscular hemoglobin; MCHC: Mean corpuscular hemoglobin concentration; MCV: Mean corpuscular volume; PLT: Platelets; C-RP: C-reactive protein.

### Antioxidant effect

#### Oxidative stress biomarkers

**Fig 4:**
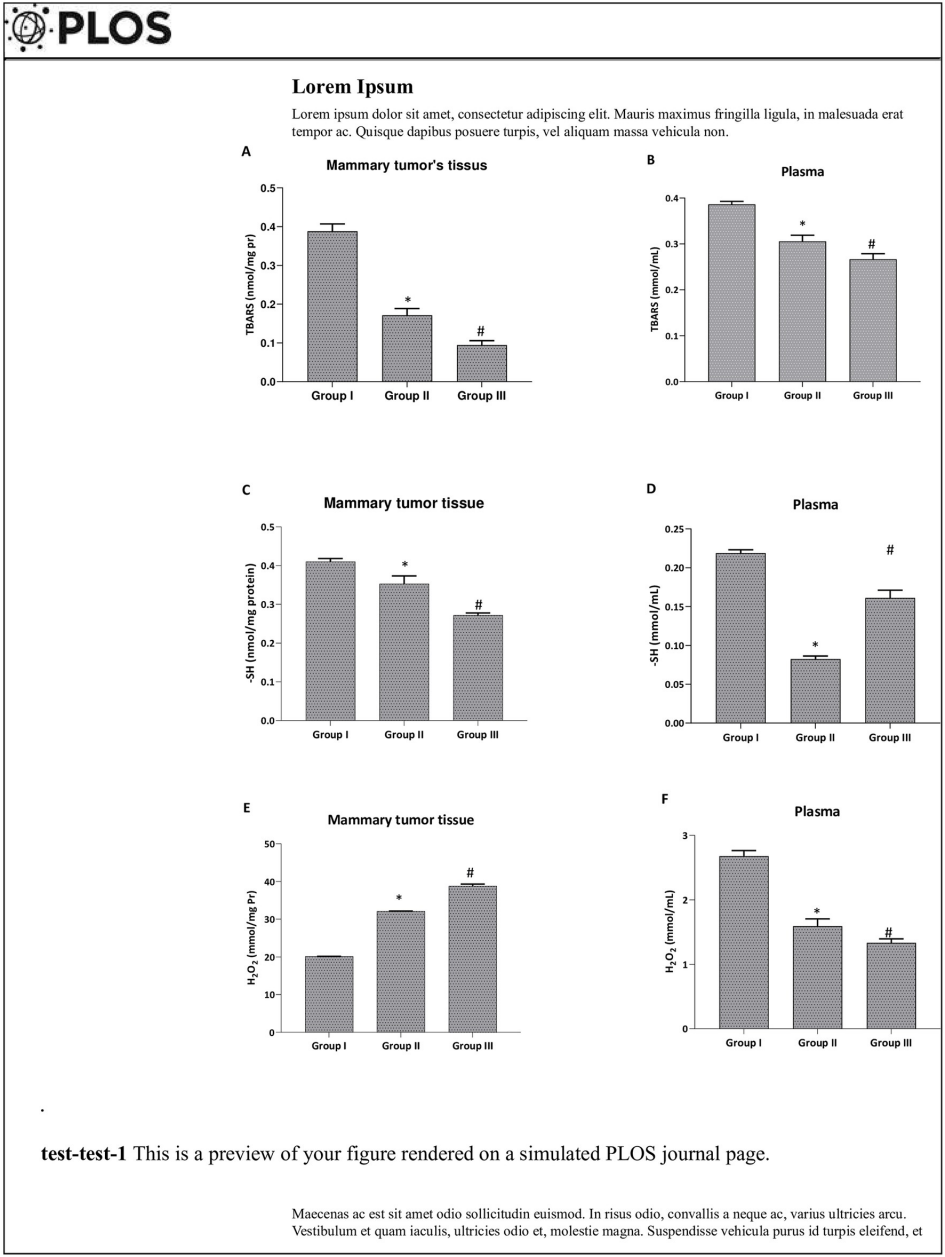
Effect of *Pistacia lentiscus* essential oil on 7,12-dimethylbenz(a)anthracene, inducing oxidative disorders in the mammary tissue and plasmaof female C57Bl/6 mice. All animals were pretreated with two doses of DMBA (20 and 50mg/kg, b.w. dose) associated with progesterone and estrogens treatments until mammary tumor induction. GroupI: had not received any treatment, while group II and III received *PL* EO (280 and 570 mg/kg, b.w. dose, respectively). Results are represented by the mean ± standard error (SEM) (n = 08). *: *p* < 0.001 vs Group I, and #: *p* < 0.05 vs Group II using non-parametric Kruskal-Wallis test.

We investigated the oxidative stress induced by DMBA (Fig 4), through the study of MDA, thiol groups and hydrogen peroxide levels in mammary tumors and plasma.

DMBA pretreatment significantly increased the MDA content in both mammary tissues and plasma (*p*<0.05) (Fig 4A and 4B) in the positive control group compared to the co-treated groups with *PL* Measurement of hydrogen peroxide levels showed a significant increase in plasma of the positive control group, while in mammary tissue levels were decreased compared to co-treated groups (Fig 4E and 4F) (*p*<0.05). We also showed a decrease in thiol groups in both mammary tissues and plasma of the positive control group (Fig 4C and 4D). However, oxidative stress was significantly reversed by co-treatments (*PL*-1 and *PL*-2), compared to the DMBA positive control group (*p*<0.05). Moreover, PL exerted a corrective effect in dose dependent manner where the used *PL*-2dose was more efficacy and corrective in the analyzed stress biomarkers.

#### Antioxidant enzyme stress biomarkers

**Fig 5:**
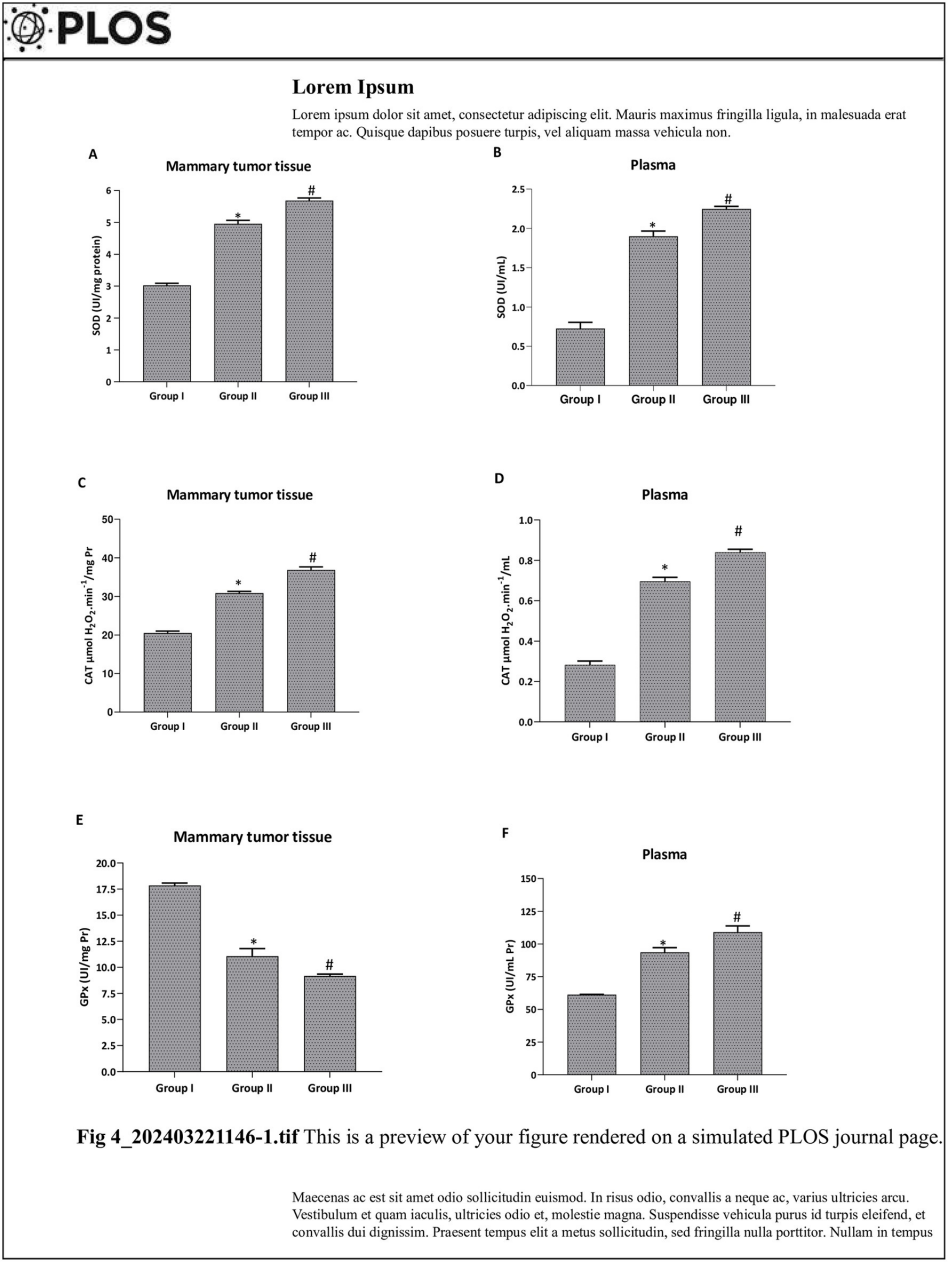
Effect of *Pistacia lentiscus* essential oil on 7,12-dimethylbenz(a)anthracene, inducing antioxidant enzyme disorders in the mammary tissue and plasma of C57Bl/6 female mice. All animals were pretreated with two doses of DMBA (20 and 50 mg/kg, b.w. dose) associated with progesterone and estrogens treatment until mammary tumor induction. Groupe I: had not received any treatment, while group II and III received *PL* EO (280 and 570 mg/kg, b.w. dose respectively). Results are represented by the mean ± standard error (SEM) (n = 08). *: *p* < 0.001 vs Group I, and #: *p* < 0.05 vs Group II using non-parametric Kruskal-Wallis test.

We investigated the response of antioxidant enzymes to oxidative stress induced by DMBA (Fig 5), through the study of the superoxide dismutase (SOD), the catalase (CAT) and the glutathione peroxidase (GPx) activities levels in mammary tissues and plasma.

We have shown that DMBA poisoning significantly reduces (p<0.05) activities of the SOD (Fig 5A) and the CAT (Fig 5C) in mammary tissues and plasma of the positive control group compared to the co-treated groups (*PL*-1 and *PL*-2). However, the GPx activity measurement (Fig 5E) was higher in the positive control group in mammary tissues while the plasmatic measurement was lower in the DMBA positive control group. Moreover, enzymatic activity in the plasma (Fig 5B, 5D and 5F) was lower significantly (*p*<0.05) in the DMBA positive control group compared to the co-treated groups and also minimized compared to mammary tissue levels. However, mice co-treated with *PL* have a corrector effect in the activity of antioxidant enzyme levels in two groups (*PL*-1 and *PL*-2).

#### Plasma scavenging capacity

Plasma was evaluated for their DPPH free radical scavenging ability. DMBA mammary cancer group showed significant decrease of the plasma scavenging activity (39.2%) compared to co-treated groups with *PL* EO (*p*<0.05). By contrast, the plasma scavenging capacity of the co-treated groups (*PL*-1 and *PL*-2) increased dose-dependently (49 and 65.3%, respectively) (Table 3). In addition, the administered dose *PL*-2 co-treated group exhibited the highest plasma scavenging activity compared to the administered dose in *PL*-1 (Table 3.) (*p*<0.05).

**Table 3:** Effect of *Pistacia lentiscus* essential oil co-treatment on DMBA mammary cancer induced disturbance in plasma antiradical scavenging capacity.

	Plasma sample ± SE		
	GI	GII	GIII
**Plasma antiradical activity**	39.2± 0.3	49± 0.6 *	65.3± 0.23 ^#^

All animals were pretreated with two doses of DMBA (20 and 50mg/kg, b.w. dose) associated with progesterone and estrogen treatment until mammary tumor induction. Group I: had not received any treatment, while group II and III received *PL* EO (280 and 570 mg/kg, b.w. dose, respectively). Results are represented by the mean ± standard error (SE) (n = 08).

*: *p* < 0.001 vs Group I, and

^#^: *p* < 0.05 vs Group II.

## Discussion

The present study aimed to explore the anti-cancer potential of *PL* EO against MCF-7 breast cancer cells *in vitro* and *in vivo* DMBA-mammary cancer on C57BL/6 mice model as well as to investigate its anti-inflammatory and antioxidant effects as implicated mechanisms. The chromatographic identification of *PL* EO bio-compounds showed a new chemotype of 39 peaks (S1 Fig in S1 File) detected in 99.32% of EO (Table 1.). The main identified compounds include monoterpenes (24.4%), Ketone (51.2%), Ester (8.6%), Sesquiterpenes (4.3%) and Hydrocarbons (1.4%) (Fig and Table in S1 File).

The main objective of this research was to evaluate the anticancer effect of *PL* EO *in vitro* against MCF-7 cell line and *in vivo* using DMBA-mammary cancer induction in female C57BL/6 mouse model. The ability of *PL* EO to inhibit human breast cancer adenocarcinoma cell proliferation was firstly assessed (Fig 1). Results showed that *PL* EO displayed antitumor effect against MCF-7 cells in dose dependent manner (Fig 1A and 1B). Our data is in agreement with previous studies (data in S1 Text), reporting an important *in vitro* anti-cancer activity of PL[9, 24]. Several studies have shown that *PL* EO reduced the PC-3 prostate adenocarcinoma cells’ growth by blocking growth in the G_l_ phase of the cycle, then suppressing the activity of NF-KB and its signaling pathway [24]. Additionally, it was shown that EO contributed to the accumulation of intracellular ROS and DNA fragmentation as confirmed by the apoptosis induced on FTC-133 thyroid carcinoma cells characterized by mutation of the tumor suppressor gene PTEN [9]. *PL* has been shown to reduce angiogenesis against fibroblasts, 13 types of human tumour/leukaemia, CRC HCT116 cell and to induce apoptosis (programmed cell death) in FTC-133 thyroid carcinoma [23, 40]. It is worth noting that PL has a potent anti-cancer activity against human alveolar adenocarcinoma, neuroblastoma, leukaemia and thyroid carcinoma [9, 24].

*In vivo*, our study investigated the ability of PL EO to reduce mammary cancer development in female C57bl/6 mouse model in standard conditions similar to human breast cancer. Overall, measurement of body weight, tumor volume, weight and burden are essential components of research using mammary cancer mouse models. Indeed, significant changes in body weight may indicate DMBA toxicity adverse effects and tumor growth (Fig 2B). Moreover, the increase in tumor volume (Fig 2C) was associated with the increase in tumor weight (Fig 2D), especially in the positive control group, which predicts tumor progression without treatment. The reduction in tumor weight in the co-treated groups was reflected in the total body weight due to the fact that we can’t weight tumors during the experiment (unlike volume measurements). This reduction was confirmed after euthanasia in “The Mean of tumor weight “. The unlimited tumor growth in the untreated group I, makes the body weight continue to increase and exceed 30g at 21^th^ week of the experiment. However, in the co-treated groups the body weight parameter began to reduce after addition of the PL treatment in 14^th^ month of the experiment (17^th^ week age). The reduction was more significant in *PL*-2 more than *PL*-1 at the end of 21^th^ week of the experiment (24^th^ week of age). Also, the tumors yield, mammary tumor volume after euthanasia, and the mean of burden (Fig 2E) allows the monitoring of the progression of the mammary tumor in the positive control group. Tacking into above the other parameters of the “mean of tumor weight”, the “tumor burden”, the “tumor volume” and the “tumor yield” (Fig 2), we found a reduction of this parameter in the co-treated groups compared to untreated group I, witch indicated the effectiveness of *PL*-1 and *PL*-2 compared to positive control (*p*<0.05). The restoration of this parameter to around normal in the co-treated groups (*PL*-1 and *PL*-2) compared to the positive control group (*p*<0.05) provides the therapeutic effect of *PL* treatment in a dose dependent manner, where *PL*-2 group was more effective (*p*<0.05).

This is the fïrst mammary induction model of hormone-dependent cancer in C57BL/6 mouse at the bases of the process of carcinogenesis initiation and the growth events promoted with progesterone and estrogen treatment until mammary tumor induction (Fig 2A). The mice preselected for this model are inbred mice of the Black-6 family (C57Bl/6J) with a depletion of the Nnt gene. They are known for their adaptive capacity in the face of chemical stress. For these reasons, they were chosen to be used for this DMBA-mammary cancer induction. It is worth noting that BC can be promoted using endogenous factors such as: age, synthetic hormone therapy and using exogenous pathogenic factors such as: smoking, exposure to pollutants and humidity-heat [41]. Research work has proven the detection of mammary tumors in rodents, after the administration of DMBA a pure carcinogen belonging to Polycyclic aromatic hydrocarbons (PAHs) family [6, 7]. The DMBA model is similar to human breast carcinogenesis, in its dependence on the presence of hormones (Fig 2A) [6]. Moreover, clinical-epidemiological studies found that a combination of hormone replacement therapy in postmenopausal women and cigarette smoke and/or pollutant (PAHs sources’) have been linked to increased BC incidence [4]. In fact, our histology results explored histo-pathological changes in mammary tissue after co-treatment with DMBA (Fig 3). The use of medicinal plants and its bio-compounds can induce significant tissue damage in DMBA-mammary cancer, similar to the damage in tumors induced by chemotherapeutic treatment of cancer (doxorubicin) [5]. Some studies have suggested that *PL* anti-cancer effects may be due to the presence of aromatic monoterpenes (Fig and Table in S1 File).like d-Limonene,.alpha.-Phellandrene, 3-carene and camphene known for their broad spectrum activities as antioxidant, anti-inflammatory [42, 43] and anticancer agents [44]. Likewise, caryophyllene, germacrene D and 4-Carvomenthenol (sesquiterpenes) (Table 1) have multiple therapeutic potentials (data in S1 Text) such as antioxidant, anti-inflammatory and anticancer activities [45, 46].

Importantly, we have tested herein *PL* EO anti-inflammatory activity and its variation on C-RP and CBCC levels (Table 2). The present study has shown that *PL* EO can reduce the levels of C-RP in the blood of co-treated groups with *PL*-1 and *PL*-2 (1.2 and 1 μg/dL, respectively) when compared to positive control group (1.96 μg/dL), proving that EO may have a potential role to decrease inflammation in rodent. C-RP is a marker of inflammation in the body, and elevated levels have been associated with a high risk of various chronic diseases [47]. In the CBCC dataset (Table 3), levels of GB, Mid PLT in the positive control group were higher compared to co-treated group, monitoring the impact of inflammation in the progression of mammary tumor, on the one hand and evaluating the efficacy of PL anti-inflammatory potential on the other hand. Lymph and Gran levels in the positive control group reflect an infection which may be due mainly to an immunodeficiency induced by the process of carcinogenesis [41]. Inflammation is a natural response of the body to injury or infection, but when it becomes chronic, it can contribute to the development of various diseases, such as arthritis, cardiovascular disease, and cancer [8, 9]. Several studies have shown that *PL* EO has potent anti-inflammatory properties, as it has been proved to attenuate pro-inflammatory mediators, such as cytokines and prostaglandins, which play a key role in the inflammatory process [48]. Cytokines are signaling molecules that play a key role in the inflammatory process, and they have been shown to be inhibited by the components of *PL* EO. Prostaglandins are another group of signaling molecules that are involved in the regulation of inflammation, and they have also been shown to be inhibited by *PL* EO [49]. *PL* involved the inhibition of the enzymes involved in cytokines and prostaglandins synthesis, such as cyclooxygenase (COX) and lipoxygenase (LOX) [48]. In addition to inhibiting pro-inflammatory mediators, *PL* has also been shown to have other anti-inflammatory effects, such as reducing oxidative stress and the activation of immune cells, and improving the overall antioxidant defense system of cells [49].

Finally, the present study has shown that *PL* EO has antioxidant activity, which is believed to be due to the presence of antioxidant bio-compounds, including phenolic bio-compounds, flavonoids, and terpenes [42, 43]. An increased oxidative stress parameter in the DMBA-group was detected. In addition, we observed decrease of H_2_O_2_ in mammary tissue as well as an increase in MDA, -SH and H_2_O_2_ levels in both mammary tissues and plasma of the DMBA-positive control group (Fig 4). Furthermore, we noted an increase of GPx activity in mammary tissue and a depletion of antioxidant enzyme activities in mammary tissues and plasma (SOD, CAT and GPx) [50]. Also, a reduction in plasma scavenging capacity has been mentioned in the same group (Table 3). The antioxidant activity of *PL* EO has also been shown to have potential therapeutic benefits in *PL*-1 and PL-2 groups. The plasma scavenging capacity was important in the *PL* co-treated groups. That is due to the plant’ ability to neutralize harmful free radicals and prevent oxidative damage to cells (Table 3). Phenolic compounds, such as caffeic acid, ferulic acid and p-coumaric acid, are known to have strong antioxidant properties, as they can effectively scavenge reactive oxygen species and prevent oxidative stress (Fig 4). Flavonoids, such as kaempferol and quercetin, and terpenes, such as terpinolene and limonene, are also known to have potent antioxidant activity [42, 43]. These chemotypes can scavenge free radicals (Table 3), protect cells from oxidative damage (Fig 4), and improve the overall antioxidant defense enzyme system (Fig 5). The combination of these antioxidant compounds in EO is believed to contribute to its potent antioxidant activity [42, 43]. The metabolism of these compounds can vary depending on the administration route and specific metabolic processes of the body. The specific mechanisms and efficacy of PL detoxification and metabolic processes require further research and one must delve into a component-by-component basis. However, there are studies that have demonstrated that PL poses no toxicity to the organism (unlike certain plants such as lavender, sage, etc.). The liver, being an indispensable organ for detoxification, stands to benefit from the supportive influence of PL. Further, a hepatoprotective effect is suggested, safeguarding the liver from potential harm and fortifying its detoxification mechanisms. Convincing evidence further indicates its potential to exert a protective influence against steatosis. Notably, dietary supplementation with PL has exhibited the capacity to mitigate the hepatic accumulation of fat, thereby augmenting overall liver function. [51]. Various PL bioactive compounds have showcased protective attributes, specifically in ameliorating carbon tetrachloride (CCl4)-induced hepatotoxicity [52]. Moreover, the plant has been substantiated for its antioxidant properties, notably demonstrated in the restoration of hepatic function following exposure to sodium arsenite [53]. PL components exhibit the capability to counteract free radicals and alleviate oxidative stress in hepatic cells. [25, 53, 54].

The rationale behind this study is that inflammation and oxidative stress are two interconnected processes that play a role in cancer development and progression [8, 9]. Both inflammation and oxidative stress can interact and exacerbate each other, leading to a positive feedback loop that promotes cancer progression [8, 9]. The mode of action of *PL* EO in DMBA-induced mouse cancer has been studied in some research but not in the mammary cancer model. The findings suggest that *PL* EO has been shown to have anti-inflammatory activity, which may reduce the risk of cancer by reducing chronic inflammation [8, 20]. Inflammation refers to a state of immune activation that is characterized by the release of cytokines and other proinflammatory mediators [41]. Chronic inflammation can contribute to the development of cancer by promoting cellular proliferation, angiogenesis, and suppression of apoptosis [41]. *PL* EO can exert its anti-inflammatory effects by several mechanisms, Firstly, anti-inflammatory activities can reduce cancer by the attenuating the pro-inflammatory cytokines production, such as TNF-α and IL-6, which have been shown to promote cancer growth and progression. Secondly, bio-compounds can inhibit angiogenesis by reducing the production of pro-angiogenic factors; the process that can support the growth and spread of cancer cells in chronic inflammation [24]. Thirdly, bio-compounds can also enhance apoptosis by promoting the activation of pro-apoptotic pathways suppressed by chronic inflammation promoting cancer progression [40]. On the other hand, oxidative stress refers to an imbalance between the cell’s ability to neutralize reactive oxygen species (ROS) and its production. ROS can cause oxidative damage to DNA, proteins, and lipids, leading to the development of cancer [21, 55]. *PL* EO can exert its anti-cancer effects by several mechanisms, including, at first, the strong antioxidant activity, which helps protect against oxidative damage and reduces the risk of cancer [8, 20]. Similarly, antioxidant activities can reduce cancer by the way of neutralizing ROS, and enhance DNA, protein and lipids repair by providing cells with the necessary tools to repair oxidative damage, which can decrease the risk of mutations and the development of cancer [55].

Bio-compounds can also inhibit oxidative stress-induced signaling pathways [56] that promote cancer growth and progression, such as MAPK- MEK/ERK and PI3K/Akt pathways [12, 57]. More importantly, *PL* has been shown to reduce cancer growth by affecting the epidermal growth factor pathway. Epidermal growth factor (EGF) is a protein involved in cell proliferation and survival, and it’s over activity has been linked to the development and progression of several types of cancer. Studies have shown that *PL* EO can inhibit the EGF-receptor (EGFR) and its downstream signaling pathways, thereby reducing cancer cell proliferation and survival [12, 57]. Additionally, EO has been shown to induce apoptosis (programmed cell death) in cancer cells, further contributing to its anti-cancer effects [8, 9, 40]. However, studies have suggested that *PL* can inhibit the EGFR and its downstream signaling pathways such as the mitogen-activated protein kinase (MAPK) and phosphatidylinositol 3-kinase (PI3K) pathways. EO has been shown to block the binding of EGF to its receptor, thus preventing activation of the downstream signaling pathway (inhibition of EGF signaling). Secondly, EO also suppresses the activity of PI3K, the enzyme that regulates cell growth and survival. By suppressing PI3K activity, the EO also blocks activation of the serine/threonine protein kinase Akt, which is involved in the regulation of cell proliferation and angiogenesis [12, 57]. Finally, *PL* EO can inhibit the EGF/PI3K/Akt pathway inducing apoptosis in cancer cells [9], leading to inhibition of tumor growth. By blocking the activation of these pathways, the *PL* can reduce the growth and survival of cancer cells [40, 57].

Importantly, *PL* anti-inflammatory and antioxidant activities can work together to reduce the risk and progression of cancer by inhibiting angiogenesis, suppressing the growth and spread of cancer cells and promoting cell death and DNA repair [23, 24].

## Conclusion

One of the more significant findings to emerge from this study is that *PL* bioactive compounds are promising sources with anti-cancer, anti-inflammatory and antioxidant activities. *PL* EO inhibits MCF-7 human breast cancer cell proliferation *in vitro*, reduces the DMBA-MC development in C57BL/6 female mice *in vivo* and has potential anti-inflammatory and antioxidant enhancements. Taken all the above study, our research highlights *PL* EO as a promising natural adjuvant for the treatment of BC. Also, targeting both inflammation and oxidative stress may represent a promising strategy for cancer prevention and treatment. Considerably, more cancer cell lines and animal model will need to be done to better characterize the precise action mechanism and the signalling pathways involved therapeutic potential benefits of *PL* molecular mechanisms, especially in the EGF pathway.

## Supporting information

S1 File Including Figure and table, chromatographic GC-MS analysis of *Pistacia lentiscus* essential oil. (PDF)

S1 Text Phytochemical analysis and biological activities of two oil-bearing extracts from fresh *Pistacia lentiscus*. (PDF)

S1 Graphical abstract (PDF)
