# Supplementary material for: Pistacia lentiscus L. revealed in vitro anti-proliferative activity on MCF-7 breast cancer cells and in vivo anti-mammary cancer effect on C57BL/6 mice through necrosis, anti-inflammatory and antioxidant enhancements
Source: PLoS One. 2024 Apr 18;19(4):e0301524. doi: 10.1371/journal.pone.0301524 (PMC11025873; doi:10.1371/journal.pone.0301524)
Supplement: S1 File — (PDF) [file pone.0301524.s001.pdf]

Print Date: 29 Nov 2018 11:44:01

Sample ID:	1HE//	Operator:	RIM	Instrument ID:	MS Instrument #1
Last Calibration:	None	Acquisition Date:	11/27/2018 4:53 AM	Data File:	...or 2611\1he--amor ext.sms
Calculation Date:	11/29/2018 11:42 AM	Method:	c:\agilentws\methode\amor ext.mth		
Inj. Sample Notes:	None				

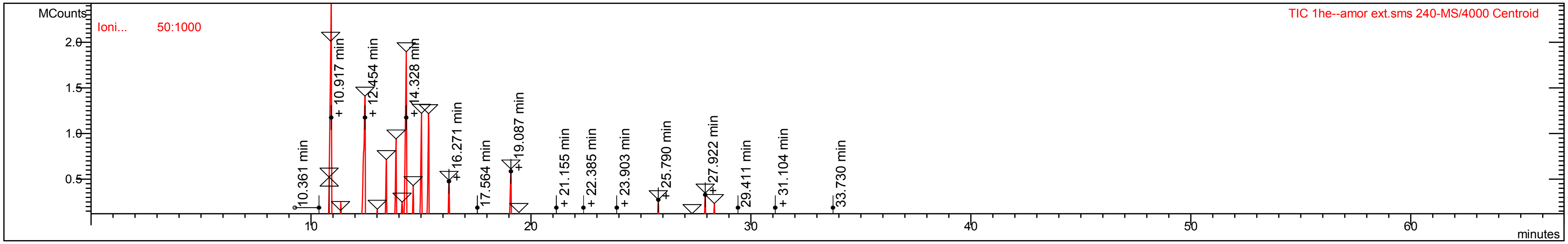

Target Compounds

#	RT	Ion	ΔRT / Actual RT	Peak Name (Actual / Specified)	Status / Range	CAS No. / Abundance	Res Type / Status	Quan Ions	Area	Amount/RF	R.Match	Group
1	10.361		0.001	3-Carene	X g	13466-78-9	Id.	93.2	32213	0.271 %	999	
2	10.631		0.000	Bicyclo[3.1.0]hex-2-ene, 2-me	X g	2867-05-2	Id.	91.2	48585	0.409 %	999	
3	10.917		0.005	(1R)-2,6,6-Trimethylbicyclo[3	X g	7785-70-8	Id.	93.2	3.138e+6	26.405 %	999	
4	11.357		0.000	Santolina triene	X g	2153-66-4	Id.	93.2	142898	1.202 %	999	
5	12.454		0.007	Cyclohexane, 1-methylene-4-(1	X g	499-97-8	Id.	93.2	2.006e+6	16.878 %	999	
6	13.012		0.006	Cyclohexane, 1-methylene-4-(1	X g	499-97-8	Id.	93.2	111157	0.935 %	999	
7	13.424		0.004	.alpha.-Phellandrene	X g	99-83-2	Id.	91.2	646618	5.441 %	999	
8	13.602		0.012	3-Carene	X g	13466-78-9	Id.	91.2	16558	0.139 %	999	
9	13.866		0.005	Cyclohexene, 1-methyl-4-(1-me	X g	586-62-9	Id.	93.2	475152	3.998 %	999	
10	14.141		0.003	tert-Butylbenzene	X g	98-06-6	Id.	119.3	346772	2.918 %	999	
11	14.328		0.004	D-Limonene	X g	5989-27-5	Id.	93.2	1.570e+6	13.211 %	999	
12	14.646		0.000	trans-.beta.-Ocimene	X g	3779-61-1	Id.	91.2	229597	1.932 %	999	
13	15.022		0.004	1,3,6-Octatriene, 3,7-dimethy	X g	3338-55-4	Id.	93.2	723896	6.091 %	999	
14	15.347		0.003	4-Terpinenyl acetate	X g	4821-04-9	Id.	91.2	924144	7.776 %	999	
15	16.271		0.002	Cyclohexene, 1-methyl-4-(1-me	X g	586-62-9	Id.	93.2	221032	1.860 %	999	
16	16.410		0.001	16.409 min, Scan: 3657	X g	None	Id.	58.1	62435	0.525 %	999	
17	16.656		0.000	1,6-Octadien-3-ol, 3,7-dimeth	X g	115-99-1	Id.	93.2	12234	0.103 %	999	
18	16.846		0.001	Butanoic acid, 3-methyl-, 3-m	X g	659-70-1	Id.	70.2	26174	0.220 %	999	
19	17.179		0.000	(E)-4,8-Dimethylnona-1,3,7-tr	X g	19945-61-0	Id.	69.2	18933	0.159 %	999	
20	17.564		0.008	2,4,6-Octatriene, 2,6-dimethy	X g	7216-56-0	Id.	121.3	18823	0.158 %	999	
21	19.087		0.004	Terpinen-4-ol	X g	562-74-3	Id.	93.2	286615	2.412 %	999	
22	19.323		0.006	11-(2-Cyclopenten-1-yl)undeca	X g	459-67-6	Id.	67.2	13350	0.112 %	999	
23	19.452		0.001	.alpha.-Terpineol	X g	98-55-5	Id.	93.2	44506	0.374 %	999	
24	20.784		0.002	Acetic acid, trifluoro-, nony	X g	30767-14-7	Id.	69.3	3287	0.028 %	999	
25	21.155		0.005	Isopentyl hexanoate	X g	2198-61-0	Id.	70.3	23056	0.194 %	999	
26	21.235		0.002	21.237 min, Scan: 3905	X g	None	Id.	71.2	7692	0.065 %	999	
27	21.837		0.007	21.830 min, Scan: 3935	X g	None	Id.	123.3	13885	0.117 %	999	
28	22.011		0.004	22.007 min, Scan: 3944	X g	None	Id.	111.3	12086	0.102 %	999	
29	22.188		0.006	Bornyl acetate	X g	76-49-3	Id.	95.2	48179	0.405 %	999	
30	22.385		0.006	2-Pentacosanone	X g	75207-54-4	Id.	58.2	63407	0.534 %	999	
31	23.338		0.000	23.338 min, Scan: 4013	X g	None	Id.	95.2	3073	0.026 %	999	
32	23.903		0.007	Camphene	X g	79-92-5	Id.	93.2	14338	0.121 %	999	
33	24.636		0.002	.alfa.-Copaene	X g	None	Id.	105.2	12021	0.101 %	999	
34	25.066		0.017	Cyclohexane, 1-ethenyl-1-meth	X g	515-13-9	Id.	91.2	10881	0.092 %	999	
35	25.790		0.000	Caryophyllene	X g	87-44-5	Id.	91.2	83253	0.701 %	999	

#	RT	ΔRT	Peak Name	Status	CAS No.	Res Type	Quan Ions	Area	Amount/RF	R.Match	Group
	Ion	Actual RT	Actual Specified	Range	Abundance	Status					
36	26.162	0.009	1-Butanol, 3-methyl-, benzoat	X g	94-46-2	Id.	105.2	25282	0.213 %	999	
37	26.545	0.000	.gamma.-Muurolene	X g	30021-74-0	Id.	105.3	6202	0.052 %	999	
38	26.642	0.002	Humulene	X g	6753-98-6	Id.	93.2	35893	0.302 %	999	
39	26.642	0.189	(-)-Aristolene	X g	6831-16-9	Id.	91.2	16510	0.139 %	906	
40	27.125	0.001	1-Isopropyl-4,7-dimethyl-1,2,	X g	16729-00-3	Id.	161.3	14106	0.119 %	999	
41	27.198	0.000	.gamma.-Muurolene	X g	30021-74-0	Id.	161.3	15150	0.127 %	999	
42	27.328	0.003	Germacrene D	X g	23986-74-5	Id.	91.2	39139	0.329 %	999	
43	27.591	0.000	Germacrene D	X g	23986-74-5	Id.	91.2	6091	0.051 %	999	
44	27.922	0.140	.alpha.-Muurolene	X g	10208-80-7	Id.	105.2	44718	0.376 %	910	
45	27.922	0.003	.alpha.-Farnesene	X g	502-61-4	Id.	93.2	115568	0.972 %	999	
46	28.112	0.000	Naphthalene, 1,2,3,4,4a,5,6,8	X g	39029-41-9	Id.	161.3	7714	0.065 %	999	
47	28.338	0.001	1-Isopropyl-4,7-dimethyl-1,2,	X g	16729-01-4	Id.	119.3	90585	0.762 %	999	
48	28.553	0.007	Cubenene	X g	29837-12-5	Id.	119.3	8567	0.072 %	999	
49	29.411	0.001	3-Hexen-1-ol, benzoate, (Z)-	X g	25152-85-6	Id.	67.3	17725	0.149 %	999	
50	30.789	0.005	.alfa.-Copaene	X g	None	Id.	119.3	7616	0.064 %	999	
51	31.104	0.005	.gamma.-Muurolene	X g	30021-74-0	Id.	161.3	8483	0.071 %	999	
52	31.373	0.012	.gamma.-Muurolene	X g	30021-74-0	Id.	161.3	3574	0.030 %	999	
53	31.986	0.008	(1R,4R,5S)-1,8-Dimethyl-4-(pr	X g	729602-94-2	Id.	119.3	3946	0.033 %	999	
54	33.730	0.009	Benzyl Benzoate	X g	120-51-4	Id.	105.2	6704	0.056 %	999	

Status and Errors:

X : Error

g : No Calibration Data. Reporting Peak Size.
