# Supplementary material for: Pistacia lentiscus L. revealed in vitro anti-proliferative activity on MCF-7 breast cancer cells and in vivo anti-mammary cancer effect on C57BL/6 mice through necrosis, anti-inflammatory and antioxidant enhancements
Source: PLoS One. 2024 Apr 18;19(4):e0301524. doi: 10.1371/journal.pone.0301524 (PMC11025873; doi:10.1371/journal.pone.0301524)
Supplement: S1 Text — (PDF) [file pone.0301524.s002.pdf]

Bull. Chem. Soc. Ethiop. **2023**, 37(6), 1487-1501. ISSN 1011-3924
© 2023 Chemical Society of Ethiopia and The Authors
Printed in Ethiopia
DOI: https://dx.doi.org/10.4314/bcse.v37i6.15 Online ISSN 1726-801X

PHYTOCHEMICAL ANALYSIS AND BIOLOGICAL ACTIVITIES OF TWO OIL-BEARING EXTRACTS FROM FRESH *PISTACIA LENTISCUS*

Omayma Abidi[1,2*], Zaineb Abdelkafi-Koubaa[3], Nadia Fares-Zagrouba[4], Salem Elkahoui[4,5], Lamjed Toumi[6], Lamjed Marzouki[7] and Ouajdi Souilem[1,8,*]

[1]Laboratory of Physiology and Pharmacology, National School of Veterinary Medicine, University of Manouba, Sidi Thabet 2020 Ariana, Tunisia
[2]Department of Biology, Faculty of Sciences of Tunis, University Tunis El Manar, 2092, Tunisia
[3]Laboratory of Biomolecules, Venoms and Theranostic Applications-LR20IPT01, Pasteur Institute of Tunis, University Tunis El Manar, 13 Pasteur Place, POB 74 Belvedere-1002, Tunisia
[4]Laboratory of Bioactive Substances-LR15CBBC03, Center of Biotechnology of Borj Cedria, University Tunis El Manar, POB 901 Hammam Lif-2050, Tunisia
[5]Department of Biology, University of Ha'il, College of Science, POB2440, Ha'il 81451, Saudi Arabia
[6]Laboratory of Sylvo-Pastoral resources, Sylvo-Pastoral Institute of Tabarka, University of Jendouba, POB 345 -8110 Tabarka, Tunisia
[7]Unit of Functional Physiology and Bio-Resources Valorization (BF-VBR), Higher Institute of Biotechnology of Beja, University of Jendouba, POB 382 Habib Bourguiba street-9000 Beja, Tunisia
[8]Biotech Pole Sidi Thabet 2020 Ariana, Tunisia

(Received June 13, 2023; Revised August 16, 2023; Accepted August 16, 2023)

ABSTRACT. This study aims to investigate the anti-cancer, antimicrobial and antioxidant activities of *Pistacia lentiscus* L. (*P. lentiscus*) extracts, as well as their phyto-chemical profile. The GC-MS crude chemical profile provided new chemotypes of fixed oil (FO) and essential oil (EO) extracts. Secondary metabolites screened high levels of polyphenols and condensed tannins in EO, while FO had the highest flavonoids content. A potent anti-proliferative activity of both extracts was shown on two human breast cancer cells (EO-IC_{50} = 5.2 mg/mL MDA-MB-231 cells). The antimicrobial activity showed that only EO was active against six multidrug resistant bacteria (inhibition zone 20-8 mm) and two fungi strains (inhibition zone 3-4 mm). Also, the highest *in vitro* antioxidant free-radical scavenging ability was in the case of EO (125.5 µg/mL). *In vivo P. lentiscus* EO provided corrective effect vs the tamoxifen (Txf) induced oxidative damage in liver and kidney in C57BL/6 in female mouse. These findings confirm that EO inhibits cell proliferation, induces bacterial death and has a potential antioxidant activity.

KEY WORDS: Phytotherapy, *Pistacia lenti*scus, Antioxidant, Antimicrobial activity, Breast cancer

INTRODUCTION

Several research programmes are on-going in all countries to find new natural bioactive compounds and develop new drugs from plants to treat human and animal diseases [1]. The goal of this global effort is to cope with the negative effects of chemical drugs on treated humans and animals, on the environment and the consumers [2]. Medicinal plants are a promising source of new drugs for treating animal and human diseases with less side-effects and costs as well as higher therapeutic index compared to chemical drugs [3, 4].

*Corresponding author. E-mail: ouajdi.souilem@biotechpole.tn
This work is licensed under the Creative Commons Attribution 4.0 International License

Pistacia lentiscus L. is a dicotyledonous plant, constituting a promising alternative source of phyto-therapeutic molecules. This species was originally from Central Asia, then spread to Mediterranean countries and USA since the first century AD. It was traditionally used as decoctions and infusions to treat wounds, eczema, skin burns, circumcisions, oral infections, diarrhea, nephrolithiasis and icterus, etc [5]. *Pistacia lentiscus* contains several bioactive compounds including terpenes, phenols, carotenoids, fatty acids and so on. These compounds are used nowadays in cosmetic, food and pharmaceutical industries as a natural antioxidant agent [6]. It is also used as synthetic intermediates, disinfectants, tanning agents, photographic developers and lubricant additives [7, 8]. Several researches have proved that *P. lentiscus* is active against neurodegenerative disorders [9], respiratory diseases (coughs and asthma…), cardiovascular and digestive diseases (peptic ulcers, haemorrhoids and so on) [10, 11]. It is worthy to note that *P. lentiscus* have a potent anti-cancer activity *in vitro* against human alveolar adenocarcinoma, neuroblastoma, leukaemia, thyroid carcinoma [12, 13] and *in vivo* activity against sarcoma and colon cancer [14].

As far as it could be ascertained, this is the first study investigating the activity of both *P. lentiscus* essential oil and fixed oil extracts in comparative study on human breast cancer cell lines. The aim of the present study was to assess the crude chemotype profiles of *P. lentiscus*' essential oil and fixed oil extracts as well as investigate the *in vitro* anti-cancer, antimicrobial and antioxidant activities.

EXPERIMENTAL

Chemicals reagents and media

The chemicals reagents were purchased from Sigma Chemical Co. (Sigma-Aldrich GmbH, Steinheim Albuch, Germany): 1,1-diphenyl-2-picrylhydrazyl radical, gallic acid, quercetin, catechin, methanol, sodium chloride, sulfuric acid, monosodium phosphate, sodium hydroxide, folin-ciocalteu reagent, carbonic acid disodium salt, vanillin, hydrogen chloride, aluminium chloride, cyclohexane, methanol, monosodium phosphate, hexaammonium molybdate, hydrogen peroxide, monopotassium phosphate, disodium hydrogen orthophosphate, lauryl sulfate sodium and tamoxifen, as well as growth media for the culturing of microbial strains such as Mueller-Hinton Broth Agar, Luria-Bertani Agar Medium, Winge-Broth, nutrient agar, agar, Trypto Caseine Soja, Sabouraud Agar, Mueller-Hinton Broth and Cancerous Cell Culture Reagents such as DMEM, penicillin-streptomycin solution EDTA, FBS, trypsin, DMSO, trypan blue and tetrazolium salt MTT (3-(4,5-dimethylthiazol-2-yl)-2,5-diphenyl tetrazolium bromide).

Plant material

Pistacia lentiscus L., (Sapindales; Anacardiaceae) was harvested from the region of Tabarka, Northeast Tunisia in different seasons at two phenological stages: drupes were bunched during the fruiting season (in December 2018), while leaves and flowers were collected during the flowering season of male flower (red colour) (in March 2018). The certified specimen was deposited in the Herbarium of the I.N.G.R.E.F-Tunisia: VS1-PL2009. Extractions were immediately made from freshly-collected plants: EO was extracted from females of *P. lentiscus* using the hydro-distillation. While, FO was extracted using the Soxhlet method. Both of *P. lentiscus* extracts were subject to GC-MS analysis.

Essential oil extraction

Hydro-distillation method was used to extract EO according to standard method [15]. Briefly, *P. lentiscus* fresh material (leaves and flowers) were put in a tank. At the boiling point (100 °C), the water vapour pressure crossed the plant leading to gasoline release accumulated on the lid. The

vapor was transformed in the refrigerant condenser from gaseous to liquid state chaining EO and flower water to the coil. EO was recovered directly without adding any solvent and stored in the dark at -20 °C until use.

Fixed oil extraction

The Soxhlet apparatus was used to extract FO according to the ISO 10520 method [16]. *P. lentiscus* fruits were macerated and put in a cellulose thimble cartridge in the extractor body, for 6 h of successive spills with reflux. Cyclohexane was used as an organic solvent. The accumulated samples in the prepared flask were subjected to a rotary evaporator (Rotary evaporator-4000-efficient Laborota) at 45 °C under vacuum. The extracted fixed oil was stored at +4 °C until use.

Gas chromatographic mass spectrometry analysis

The crude chemical profiles of *P. lentiscus* EO and FO were performed using a gas chromatography coupled to mass spectrometry system (GC-MS) on Agilent HP 7890 (II) gas chromatography instrument coupled with an Agilent HP 5975 mass spectrometer (Agilent, CA, USA). The separation was accomplished in a HP-5MS capillary column (30 m×0.250 µm film thickness, Agilent Technologies, Hewlett-Packard, CA, USA). Helium (99.99 %) was used as carrier gas, released at a constant flow rate of 1.2 mL/min; a split ratio of 60:1; mass range of 4 - 300 m/z; scan time of 1 s. The initial oven temperature started at 40 °C, and then increased 5 °C/min to 280 °C. The injector temperature was set at 280 °C. The detection was made in full scan mode for 60 min. Mass spectrometry (MS) operating parameters were as follows: ion source temperature: 200 °C, interface temperature: 280 °C, ionizing electron energy (EI) mode: 70 eV, scan range: 50–1,000 m/z. EO and FO bio-compounds' interpretation and identification were performed by comparing mass spectra with those referenced in the Wiley 09 database, NIST 2011 Mass Spectral Database library of the GC-MS data system.

Phytochemical screening of secondary metabolites

Total phenolic content (TPC) was estimated according to the Folin-Ciocalteu method [17]. Briefly, 1 mL of the Folin-Ciocalteu reagent (10 mg/mL) was added to 50 µL of *P. lentiscus* extracts, then mixed with 5 mL of aqueous Na_2CO_3 (20 g/L). The reduction gave a dark blue coloration of the solution that was quantified at 760 nm wavelength and compared to the blank. The calculation of TPC was performed referring to gallic acid (GA) standard calibration curve. The concentration was estimated as milligram GA equivalent per gram dry weight (mg GAE/g DW).

Flavonoids content (FC) was estimated using aluminium chloride colorimetric technique [17]. Briefly, 0.5 mL of *P. lentiscus* extracts and 0.5 mL of chloride reagent ($AlCl_3$, 10 mg/mL) were mixed with 2 mL of sodium acetate ($C_2H_3NaO_2$). Flavonoids formed yellowish complexes after aluminium chelation, the absorbance was measured at 430 nm wavelength and compared to the blank. The concentrations of flavonoids were deduced from a standard calibration curve and expressed as milligram Qt equivalent per gram dry weight (mg QtE/g DW).

Condensed tannins content (CTC) was estimated with the vanillin method in an acidic medium [18]. *Pistacia lentiscus* extracts (0.05 mL) were added to 3 mL of vanillin solution (4 g/L) then mixed with 1.5 mL of hydrochloric acid (HCl). The mixture produced a specific red colour that was quantified at 500 nm wavelength and compared to the blank, referring to catechin (Ct) standard calibration curve. The concentration of CTC was expressed as milligrams Ct equivalent per gram of dry weight (mg CtE/g DW).

Pistacia lentistucs anti-proliferative activity

MDA-MB-231 and MCF_7 cell lines were seeded in 96-well culture plates at a concentration of 10^4 cells /well. After that, cells were incubated alone or plus increased concentrations of *P. lentiscus* extracts. After either 24 h or 72 h treatment incubation, cellular viability and proliferation were assessed using the 3-(4,5-dimethlthiazol-2-yl)-2,5-diphenyltetrazolium bromide tetrazolium reduction colorimetric MTT assay [19]. Cells were fixed with 3.7% formaldehyde, then stained with 0.1% crystal violet, and finally lysed with SDS. Absorbance was measured with a microplate reader (Multiskan, Lab systems, GmbH) at 560 nm wavelengths. The untreated cells, containing only medium, were used as a positive control. The results were expressed as percentages of viable cells compared to non-treated cells taken as controls.

Pistacia lentiscus antimicrobial activity

A total number of six strains were used: multidrug resistant (MDR) bacteria strains of three Gram-positive bacteria: *Enterococcus faecalis* (ATCC 29212), Methicillin-resistant *Staphylococcus aureus* (MRSA, ATCC 6538), *Listeria monocytogenes* (ATCC 19195) as well as three Gram-negative bacteria: *Pseudomonas aeruginosa* (ATCC 2134), *Escherichia coli* (ATCC 35218), *Salmonella enteritidis* hospital strain (DMB 560) and three fungi strains *Candida albicans* (ATCC 1024) *Rhizoctonia solani (RS5.2)* and *Fusarium solani* were kindly provided by the Laboratory of Bioactive Substances, Center of Biotechnology of Borj Cedria, Tunisia.

The potential antimicrobial activity against bacterial and fungal reference strains of *P. lentiscus* extracts was screened using the agar well diffusion method [20, 21]. Bacteria and yeast strains were cultured in Luria-Bertani Agar (LB) and Winge-Broth (WB) media, respectively, and incubated at 30 °C for 24 h. The supernatant was collected in saline (0.9 g/L) at an approximate concentration of 10^8 CFU/mL. Wells of 6 mm in diameter were punched in agar plates medium and filled with 100 µL of *P. lentiscus* extracts. Samples were allowed to diffuse at 4 °C for 2 h, then at 37 °C and 35 °C for bacteria and fungi, respectively, for 24 and 72 h. The antimicrobial activity was evaluated by measuring inhibition zones. The minimum inhibitory concentration (MIC) of *P. lentiscus* extracts on different bacterial strains was estimated by the liquid microbroth dilution method [21] using an ELISA plate for the phase of half-logarithmic growth. A dilution series of the oils was carried out and bacterial cell suspensions (10^8 CFU) were added in LB medium. After incubation of the plates at 37 °C for 24 h, bacterial growth was recorded using a microplate reader (Bioteck, ELx 800) at 600 nm wavelengths. Minimum inhibition concentration (MIC) was defined as the lowest concentration that induced a significant decrease in inoculums viability growth higher than 90%.

Pistacia lentiscus in vitro free radical scavenging capacity

P. lentiscus EO and FO extracts were subjected to 2,2-diphenyl-1-picryl-hydrazyl-hydrate (DPPH) free radical scavenging method [17] and the DPPH radical reduction was estimated at 516 nm wavelength compared to the methanol used as blank. The inhibition (%) of the DPPH free radical scavenging ability was measured as a function of extracts concentrations as follows:

$$\text{Scavenging effect (\%)} = 100\text{x} [(\text{Abs}_{\text{control}} - \text{Abs}_{\text{sample}}) / \text{Abs}_{\text{control}}]$$

where: $\text{Abs}_{\text{control}}$: absorbance of pure DPPH and $\text{Abs}_{\text{sample}}$: absorbance of samples

Pistacia lentiscus in vitro antioxidant activity

Adult C57B/6 female mice (25–30 g) were divided into 4 groups of 10 animals in each: Group I (CTR): animals served as control and received equivalent volume of H_2O + NaCl (1 mL) every day for 1 month, group II (CTR^+): animals were treated daily with tamoxifen (Txf) (60 mg/kg b.w.) for 1 month, group III (CTR^++*P. lentiscus*): animals received Txf and *P. lentiscus* EO (40

mg/kg b.w.) for 4 weeks in the same conditions, group IV (*P. lentiscus*): animals received *P. lentiscus* EO (40 mg/kg b.w.) for 1 month in the same conditions. Kidney and liver were used to detect oxidative stress biomarkers: lipid peroxidation was measured by the detection of level of malondialdehyde (MDA) content according to standard method [22]. Thiol groups (-SH) concentration in both organs was performed according to a standard method [23]. The determination of enzymatic antioxidant activities was accomplished by detection of the superoxide dismutase enzyme SOD activity according to a standard method [24] with slide modifications. The catalase activity CAT was measured according to a standard method [24].

Statistical analysis

Data was analysed using GraphPad Prism 8.4.2 Software (La Jolla, CA, USA). Data were determined by one-way ANOVA, followed by Tukey's post hoc test and was expressed as the mean ± standard error (SE) $p < 0.05$ was considered significant.

RESULTS AND DISCUSSION

Our present work determined the phyto-chemical composition of EO and FO of *P. lentiscus* extracts. We then tested their anti-cancer, anti-microbial and antioxidant activities.

Gas chromatographic mass spectrometry analysis

P. lentiscus extracts were subject to GC-MS analysis. The EO chromatographic identification has depicted 19 piks of volatile compounds in 99.97% of EO (Table 1; Figure 1A). The main bio-compounds were alpha-pinene (28.92%), beta-phellandrene(19.95%), beta-pinene (13.38%), gamma-terpinene (8.12%) and ocimene (7.13%). The FO crude chemical profile has depicted six piks of fatty acids in 100% of FO (Table 2; Figure 1B). The main bio-compounds were beta-pinene (53.09%), sabinene (53.09%) and camphene (19.55%).

Table 1. Gas chromatographic mass spectrometry analysis of *Pistacia lentiscus* L. essential oil.

Peaks	Ret time	Area	% of total	ID
1	6,128	52793770	28.92%	alpha-Pinene
2	6,486	1048557	0.57%	Camphene
3	7,074	5970424	3.27%	Sabinene
4	7,167	24413945	13.38%	beta-Pinene
5	7,478	694963	0.38%	β-Myrcene
6	7,849	7179609	3.93%	l-Phellandrene
7	8,169	10313956	5.65%	alpha-Terpinene
8	8,377	2478338	1.36%	*p*-Cymene
9	8,498	32769265	17.95%	beta-Phellandrene
10	8,705	2360146	1,29%	(1R)-2,6,6-Trimethylbicyclo (3.1.1)heptane
11	8,989	13008262	7.13%	Ocimene
12	9,299	14820787	8.12%	gamma-Terpinene
13	10,115	3676760	2.01%	α-Terpinolene
14	10,187	863731	0.47%	2-Nonanone
15	12,602	6514287	3.57%	Terpinen-4-ol
16	15,733	1013152	0.56%	2-Undecanone
17	19,124	632738	0.35%	trans-Caryophyllene
18	21,209	1047756	0.57%	α FARNESENE
19	21,645	923171	0.51%	δ-Cadinene

Compounds are listed in order of their elution on HP-5MS capillary column. Interpretation and identification were based on a comparison of the compound's mass spectral data and Koyats retention indices (RI) with those of NIST 2011 Mass Spectral database Library.

Table 2. Gas chromatographic mass spectrometry analysis of *Pistacia lentiscus* L. fixed oil.

Peaks	Ret time	Area	% of total	ID
1	6.128	52793770	1.58%	Palmetic acid
2	6.486	1048557	19.55%	Camphene
3	7.074	5970424	22.56%	Sabinene
4	7.167	24413945	53.09%	beta-Pinene
5	7.478	694963	1.93%	β-Myrcene
6	7.849	7179609	1.29%	l-Phellandrene

Compounds are listed in order of their elution on HP-5MS capillary column. Interpretation and identification were based on a comparison of the compound's mass spectral data and Koyats retention indices (RI) with those of NIST 2011 Mass Spectral database Library.

The crude chemical profile proved richness in bio-active compounds in EO and FO extracts. Composition analyses of secondary metabolites revealed important levels of phenols and condensed tannins contents in EO as well as high levels of flavonoïds in *P. lentiscus* FO.

Phytochemical screening of Pistacia lentiscus extracts

Secondary metabolites were presented in (Figure 2.A). Essential oil had the highest contents in TPC and CTC (166.2 ± 0.07 mg GAE/g DW and 50.7 ± 0.09 mg Ct E/g DW, respectively). Compared to EO (30 ± 0.21 mg QtE/g DW), FO had a higher FC concentration (70 ± 0.02 mg QtE/g DW).

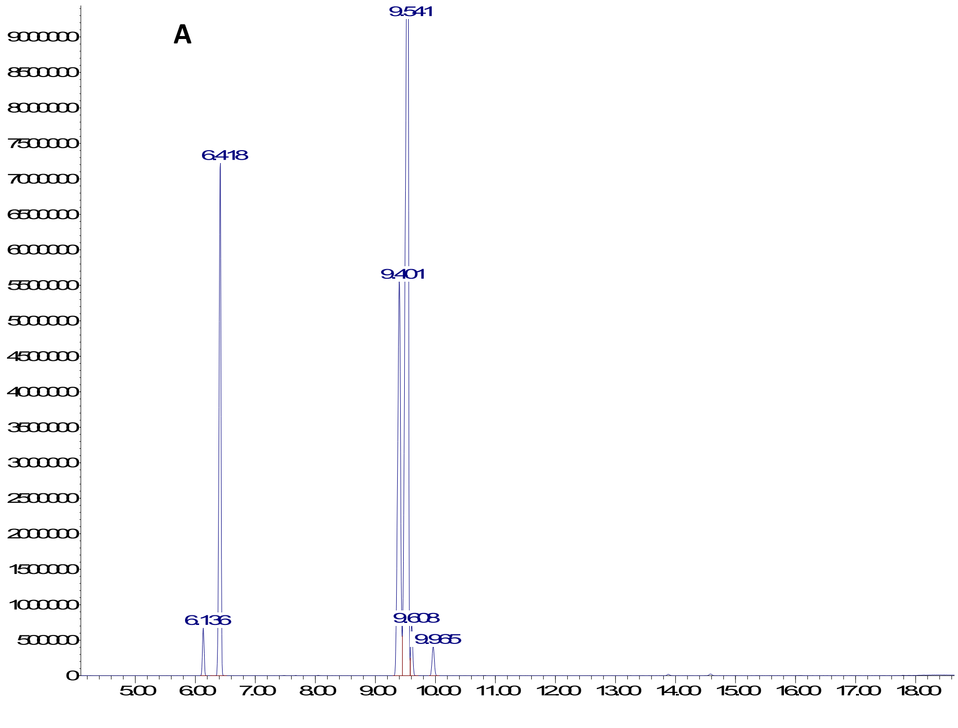

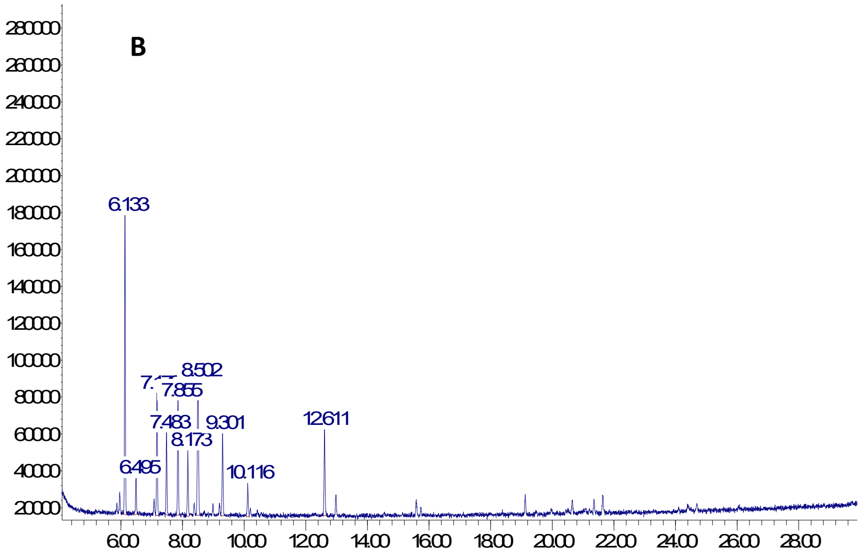

Figure 1. Chromatographic profile of *Pistacia lentiscus* L. fixed oil extracted from drupes using soxhlet method (A) and essential oil extracted from leaf and male flower using hydro-distillation method (B).

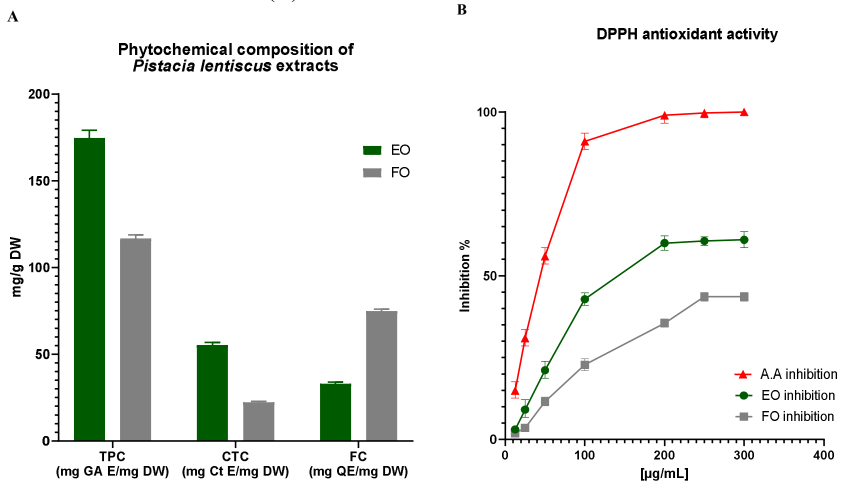

Figure 2. Phytochemical composition of secondary metabolite compounds in essential oil and fixed oil of *Pistacia lentiscus* (1A) and the dose-response of the DPPH free radical scavenging antioxidant capacity (1B). CTC: condensed tannins content; FC: flavonoids content; TPC: total phenolic content; mg Ct E/g DW: expressed as milligram catechin equivalent per gram dry weight; mg GA E/g DW: deduced as milligram gallic acid equivalent per gram dry weight; mg QE/g DW: expressed as milligrams quercetin equivalent per gram of dry weight. AA: ascorbic acid; EO: Essential oil; FO: fixed oil; DPPH: 1,1-diphenyl-2-picrylhydrazyl. Data are expressed as mean ± standard error (SE) (n = 3).

Our results are in agreement with research proving that extracts from leaves and flowers have the highest content of phenolic compounds compared to other parts like fruits, barks and roots [8, 25, 26]. Several studies have proven that hydro-distillation is the most sophisticated extraction method compared to other EO extraction techniques. However, Soxhlet facilitates the material transfer without degradation at high temperature but does not allow extraction of phenols like during the hydro-distillation [27, 28]. Gallic acid, catechin and quercetin were used as reference molecules to dose phenols, condensed tannins and flavonoïds, respectively. These reference molecules do not reflect the real content of the same family compounds, like caffeic acid in phenols (GA), kaempferol and myrecitin in flavonoids (Qst). Our results proved that phenolic compounds concentrations vary according to the part of the plant, the physiological stage, extraction methods and the used solvents [25].

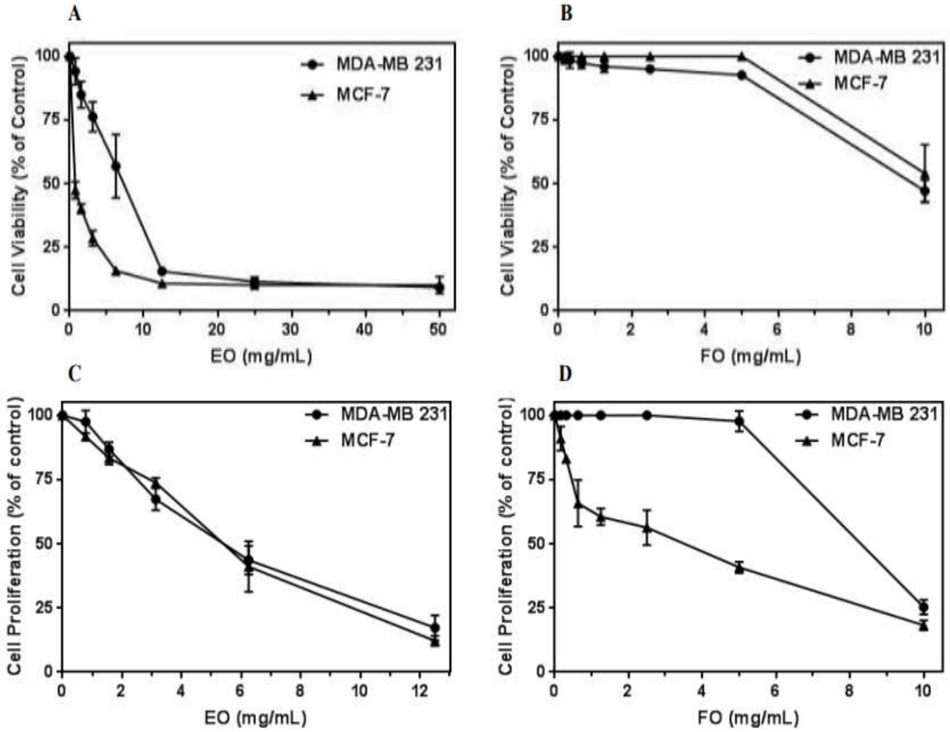

Figure 3. Effect of *Pistacia lentiscus* extracts on the viability (24 h) and cell proliferation (72 h) of MCF-7 and MDA-MB-231 cells. MCF-7 cells were treated with essential oil extracted from female flowers while MDA-MB-231 cells were treated with essential oil extracted from male flowers (A and C, respectively). Both of the used cells were also tested with extracted fixed oil from drupes (B and D). Data represent the mean ± standard error of mean (SEM) of two independent experiments performed in triplicates.

Pistacia lentiscus anti-cancer activity

The significant findings of this research were to evaluate the ability of *P. lentiscus* extracts to decrease cell proliferation on human breast cancer adenocarcinoma. *P. lentiscus* extracts reduced cell viability in a dose-dependent decrease in MDA-MB-231 and MCF-7 cells viability from the first 24 h (Figure 3 (A, B). Both *P. lentiscus* EO and FO reduced cell proliferation after 72 h of

treatment in a dose-dependent manner (Figure 3(C, D)). Interestingly, *P. lentiscus* EO showed an anti-proliferative activity on MDA-MB-231 cells (IC_{50} = 5.2 mg/mL). On the other hand, FO displayed anti-proliferative effects on MDA-MB-231 and MCF-7 cells in dose dependent manner with IC_{50} values of 8.5 and 3.5 mg/mL, respectively.

Our findings are in agreement with studies reporting that *P. lentiscus* extracts blocks the differentiation of cancerous cells and fragmentation of inter-nucleosomal DNA against fibroblasts and 13 types of human tumour/leukaemia cells. The same phenomena was reported in CRC HCT116 cell lines by inhibition of the p53 and p21 proteins with growth inhibitory effect via G1 phase cell cycle arrest [14, 29]. Likewise, decoction of *P. lentiscus* dried roots was active against gastric cancer. Moreover, *P. lentiscus* was proven to be efficient against *Helicobacter pylori*, the implicated germ in gastric cancer [30].

Pistacia lentiscus antimicrobial activity

We tested herein multidrug bacterial strains involved in nosocomial disease, food poisoning, endocarditis, osteomyelitis, skin infections, pneumonia and sometimes urinary tract infections. These strains were only sensitive to EO of *P. lentiscus* with the highest inhibition zone diameters recorded against *E. faecalis* (20 mm), followed by *S. enteritidis* (16 mm), *L. monocytogenes* (15 mm) and *S. aureus* (12 mm). The medium activity with lower zone of inhibition zone was recorded against *P. aeruginosa* and *E. coli*. In the fungi strains data, low activity was detected against fungi *Rhizoctonia solani (RS5.2)* (04 mm) and *Fusarium solani* (03 mm) (Table 1). However, *P. lentiscus* EO and FO extracts showed no inhibitory activity against the tested *C. albicans* strain. *P. lentiscus* proved an important activity against *E. faecalis*, *S. enteritidis*, *L. monocytogenes* and *S. aureus* and the medium activity against *P. aeruginosa* and *E. coli*. While the lowest activity was recorded with *Fusarium solani* and *Rhizoctonia solani (RS5.2)* yeast strains.

Table 3. Diameters of inhibition halos of microbial growth for essential oil and fixed oil of *Pistacia lentiscus.*

Microbial strains	Inhibition zone diameter (mm)	
	EO ± SE	FO ± SE
Gram-positive bacteria		
Enterococcus faecalis ATCC 29212	20± 0.5	00
Staphylococcus aureus ATCC 6538	12± 0	00
Listeria monocytogenes ATCC 19115	15± 0.5	00
Gram-negative bacteria		
Salmonella enteritidis DMB 560	16± 0	00
Pseudomonas aeruginosa ATCC2134	08± 0.5	00
Escherichia coli ATCC 25922	08± 0	00
Fungi strain		
Candida albicans ATCC 1024	00	00
Rhizoctonia solani (RS5.2)	04± 0	00
Fusarium solani	03± 0.5	00

Values were expressed as mean ± standard deviation (n = 3), ($p < 0.05$). EO: Essential oil; FO: Fixed oil; ATCC: American Type Culture Collection; SE: Standard error.

Previous studies showed that among different *P. lentiscus* extract types, only EO was effective against gram-positive and negative bacterial strains [7, 8]. Similarly, our study confirmed the absence of any *P. lentiscus* FO activity. In Algeria, Chrysoula *et al.* reported a FO bactericidal activity against three bacterial strains involved in skin and respiratory tract infections (*Aeromonas hydrophila*, *Staphylococcus aureus* and *Klebsiella pneumoniae*). The same study showed no antibacterial activity of FO for the other six tested districts. This difference could be due to the fact that the plant prevails in different geo-climatic conditions [8, 31]. The MIC was around 1 mg/mL for *E. faecalis* and *S. aureus*. For the other bacteria strains, MIC was higher than 1 mg/mL (Table 2).

Table 4. Minimal inhibitor concentration of the active extract.

Bacterial strains	EO
	MIC (mg/mL)
Enterococcus fecalis ATCC 29212	1
Staphylococcus aureus ATCC 6538	1
Listeria monocytogenes ATCC 19115	1.2
Salmonella enteritidis DMB 560	1.125
Pseudomonas aeruginosa ATCC 2134	1.6
Escherichia coli ATCC 25922	1.75

Values were expressed as mean ± standard deviation (n = 3), ($p < 0.05$). EO: Essential oil; MIC: Minimal inhibitor concentration.

MIC EO of *P. lentiscus* values were approximately 1 for *E. faecalis* and *S. aureus*. For the other bacteria stains, MIC was higher than 1 mg/mL (Table 2). The EO antibacterial activities were lower than those of antibiotic discs like ampicillin sodium, and streptomycin sulfate. *Staphylococcus aureus* was resistant to methicillin and *S. enteritidis* was resistant to cefotaxime and teicoplanine. However, *P. lentiscus* EO was active against these strains. Cefotaxime and teicoplanin have a MIC higher than 1 mg/mL against *S. aureus* and *L. monocytogenes*, while EO had a MIC approximately 1 mg/mL against *S. aureus*. Further, amoxicillin had a high MIC (4 µg/mL) for *E. coli*, *S. aureus* and *S. enteritidis* compared to EO [32].

Pistacia lentiscus in vitro free radical scavenging capacity

Different concentrations of *Pistacia lentiscus* EO and FO extracts were subjected to neutralize DPPH• free radicals (Figure 2B). Ascorbic acid revealed a maximum DPPH activity of 100%, while *P. lentiscus* EO and FO showed 60 and 43.78% of activity, respectively presented in Figure 2B. Ascorbic acid exhibited the highest DPPH radical scavenging activity followed by EO and FO, with IC_{50} of 58.01 ± 0.92, 125.55 ± 0.28 µg /mL and 288.1 ± 0.69 µg/mL, respectively.

Our data are in agreement with previous studies reporting an important antioxidant activity of *P. lentiscus* EO *in vitro* [27, 33, 34]. Antioxidant activity of various parts of *P. lentiscus* proved the greatest activity with aqua solvent in leaves and twigs extracts more than hexane extracts [35]. However, our result showed an important scavenger effect of EO: the hydro-distilled extract compared to the results reported by Charef *et al.* and Belyagoubi *et al.* [36, 37]. The ability of *P. lentiscus* extract to neutralize DPPH• radicals was dose-dependent and is probably due to the presence of hydroxyl group in the phenolic compounds serving as electron donors to neutralize the DPPH• radical.

Pistacia lentiscus in vitro antioxidant activity

We investigated the oxidative stress induced by Txf (Figure 5), via the study of the activity of antioxidant enzymes in liver and kidney levels in C57B/6 female mice. We have shown that Txf

treatments significantly reduced catalase (Figure 5A) activity and increased SOD (Figure 5B) activity at the organ levels. However, co-treatment with *P. lentiscus* has a corrector effect. Txf treatment significantly increased MDA levels in both organs (Figure 5 .C). The induced lipid peroxidation was significantly reversed by *P. lentiscus.* We have also shown a decrease in thiol groups in the group treated with inverted Txf after treatment with *P. lentiscus* EO (Figure 5.D). *P. lentiscus* EO exerted a corrective effect on oxidative stress parameters.

Our data are in agreement, with previous studies reporting an important antioxidant activity of EO of *P. lentiscus* [27, 33, 34]. Moreover, Klibet *et al.* have proven an antioxidant effect demonstrated *in vivo*, through the retraction of hepatic function after intoxication with sodium arsenite [38, 39].

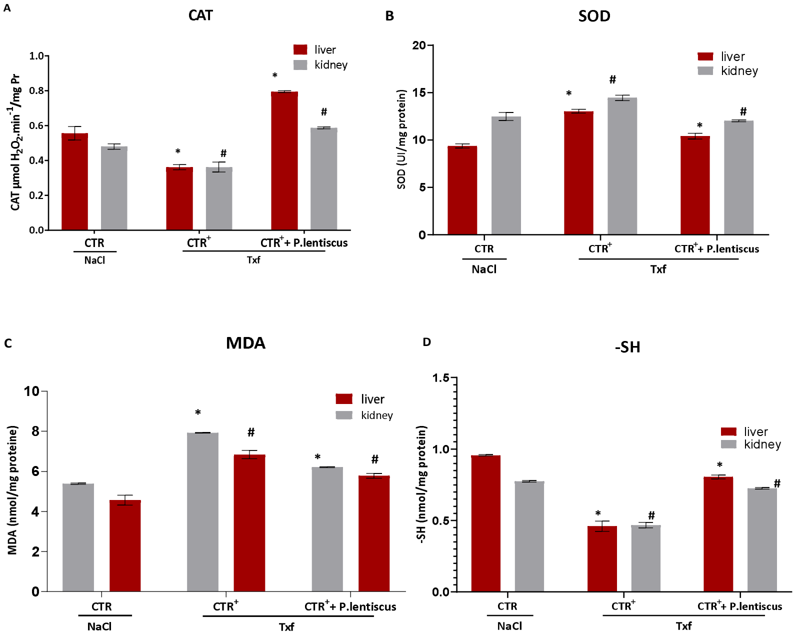

Figure 5. Effect of *Pistacia lentiscus* essential oil on tamoxifen, inducing oxidative disorders in the liver and kidneys of C57/B6 mice. Enzymatic antioxidant activity: CAT (A) and SOD (B). Stress biomarkers: MDA (C) and -SH (D).

Animals were pretreated with daily dose of Txf (80 mg kg, p.c., p.o.) alone for the (CTR^+) group and with addition of essential oil of *P. lentiscus* (40 mg kg, p.c., p.o.) in the (CTR^+ + *P. lentiscus*) group. The (CTR) group was treated with (0.9% NaCl). Results are represented by the mean ± standard error (SE) (n = 10). *: $p < 0.001$ vs control, and #: $p < 0.05$ vs Txf using non-parametric Kruskal-Wallis test.

One of the reasons behind this study is that some bacteria can be linked to several disorders in oxidative stress, proliferation signaling pathway and membrane receptors, leading to chronic diseases like hypertension, diabetes, neurodegenerative diseases and cancer [9, 38]. Furthermore, each year 2.2 million new cases of cancer are thought to result from infection with pathogens [40]. Several researches have proved that same bacterial communities can also be found abundantly in cancerous tissue, and be significantly correlated with the expression of genes involved in the pathways of proliferation, metastasis and stimulate the oncogenic process [41].

Moreover, it is known that the antibacterial and antifungal activities depend on the nature of the phenol and the presence of biologically active compounds. Some bio-compounds belong to the class of terpenes, flavones, flavonols, alkaloids and phenylpropanoids, demonstrated promising antimicrobial activity especially through cell membrane disruption especially alpha-pinene, limonene maslinic acid and oleic acid [1]. These compounds act as destabilizers of the cytoplasmic membrane by leaking K+ from the cytosol while functioning as a proton exchange which causes the pH gradient across the membrane to decrease. Therefore, the collapse of the proton motive force and the depletion of ATP that leads to the cell death of the bacterium [42-44]. A combination of bio-compounds with traditional antibiotics has also shown that polyphenols have a synergistic effect with ciprofloxacin, gentamicin and tetracycline in relation to *MDR K. pneumonia*. Flavonoids in turn have a synergistic effect with penicillin and amoxicillin, against *MDR S. epidermidis*. Terpenes have also been proven to synergize with gentamicin against *MRSA* and *MDR K.pneumoniae*. The potential antimicrobial effect of these bio-compounds was respectively based on inhibition of efflux pump and biofilm, inhibition of peptidoglycan synthesis, β-lactamase activity, decrease of fatty acids and increased cytoplasmic membrane permeability [42-44]. Phenolic hydroxyl groups and sugars in the case of flavonoids seemed to have a crucial role [1].

Studies have proven an antioxidant effect of *P. lentiscus* demonstrated *in vitro* and *in vivo*, to prove the protective effect against oxidative stress implicated in chronic disease such as cancer, HTA, diabetes, Alzheimer, Ect [38]. Furthermore, phenolic compounds serve as protective agents against the oxidative degradation of carbohydrate lipids and protein in particular that of DNA [6]. Oxidative stress results in abnormal circumstances at the cellular and tissue level, including proliferation disorders, the immune system, gene expression and membrane receptors. [45].

Antioxidant activity is strongly correlated to a high content of phenolic compounds and the nature of these bioactive compounds [37]. Similar studies revealed that plants have "phytoprotant" mechanisms acting in association or in synergy with phenols to inhibit oxidative stress. Some of them are non-enzymatic molecules like vitamins C and E, carotenoids, albumin, uric acid, polyamines and some others are enzymes-like molecules (catalase, superoxide dismutase and glutathione peroxidase) [46-48]. Phyto-therapeutic products and nutritional supplements with bioactive phenols could be a good food adjuvant [49, 50]. It can be also exploited as a preservative in food and in treatment of infectious diseases [7]. Besides, the protective effect of *P. lentiscus* extracts is referred to as phytoanticipins and phytoprotant [48].

CONCLUSION

In the present study, the phyto-chemical screening of *P. lentiscus* revealed the presence of various bio-compounds including secondary metabolites. Importantly, the in vitro study showed a dose-dependent anti-proliferative activity of EO on MDA-MB-231 and MCF-7 breast cancer cell lines. More importantly, the antimicrobial activity evaluation showed that only EO was active against the six tested bacteria and two fungi strains. In addition, EO was the highest antioxidant free-radical scavenging extract compared to FO. Also, *P. lentiscus* EO has proved an antioxidant potential on Txf induced oxidative damage in the liver and kidney of C57/BL6 in female mice. Our main finding showed that EO is promising sources with anti-human breast cancer, antibacterial and antioxidant activities. Moreover, we attend to test other cancer cell lines such as uterine cancer cells, prostate (PC-3), non-small cell lung cancer (A459), neuroblastoma and melanoma, to better understand the different mechanisms and signaling pathways involved in the mode of action of *P. lentiscus.*

ACKNOWLEDGEMENTS

We are grateful to Ministry of Higher Education and Scientific Research of Tunisia and its institutes: the National School of Veterinary Medicine of Tunisia, the Higher Biotechnology

Institute of Biotechnology of Beja-Tunisia, the Center of Biotechnology of Borj Cedria-Tunisia and the Sylvo-Pastoral Institute of Tabarka-Tunisia. We are grateful to all of these institutions who allowed us access to the laboratory and the use of needed equipment. We thank Prof. Fatma GHARBI (Faculty of Sciences, Univ. Tunis El Manar, Tunisia), Pr. Mohamed GHARBI (National School of Veterinary Medicine, University of Manouba, Tunisia) and Dr. Ghassan TAYH (Faculty of Sciences, University of Tunis El Manar, Tunisia) for their encouragement and their technical advice.

REFERENCES

1. Álvarez-Martínez, F.; Barrajón-Catalán, E.; Herranz-López, M.; Micol, V. Antibacterial plant compounds, extracts and essential oils: An updated review on their effects and putative mechanisms of action. *Phytomedicine* **2021**, 90, 153626.
2. Davis, C.; Naci, H.; Gurpinar, E.; Poplavska, E.; Pinto, A.; Aggarwal, A. Availability of evidence of benefits on overall survival and quality of life of cancer drugs approved by European Medicines Agency: Retrospective cohort study of drug approvals 2009-13. *BMJ* **2017**, 359.
3. Panikar, S.; Shoba, G.; Arun, M.; Sahayarayan, J.J.; Nanthini, A.U.R.; Chinnathambi, A.; Alharbi, S.A.; Nasif, O.; Kim, H.-J. Essential oils as an effective alternative for the treatment of COVID-19: Molecular interaction analysis of protease (Mpro) with pharmacokinetics and toxicological properties. *J. Infect. Public Health* **2021**, 14, 601-610.
4. Sun, Y.; Qian, J. Botanical drug clinical trial: Common issues and future options. *Acta Pharm. Sin. B* **2021**, 11, 300-303.
5. Pachi, V.K.; Mikropoulou, E.V.; Gkiouvetidis, P.; Siafakas, K.; Argyropoulou, A.; Angelis, A.; Mitakou, S.; Halabalaki, M. Traditional uses, phytochemistry and pharmacology of Chios mastic gum (*Pistacia lentiscus* var. Chia, Anacardiaceae): A review. *J. Ethnopharmacol.* **2020**, 254, 112485.
6. Feng, X.-H.; Zhang, Y.; Wu, R.-Y.; Derynck, R. The tumor suppressor Smad4/DPC4 and transcriptional adaptor CBP/p300 are coactivators for Smad3 in TGF-β-induced transcriptional activation. *Genes Dev.* **1998**, 12, 2153-2163.
7. Pachi, V.; Mikropoulou, E.; Dimou, S.; Dionysopoulou, M.; Argyropoulou, A.; Diallinas, G.; Halabalaki, M. Chemical profiling of *Pistacia lentiscus* var. Chia resin and essential oil: ageing markers and antimicrobial activity. *Processes* **2021**, 9, 418.
8. Moeini, R.; Memariani, Z.; Asadi, F.; Bozorgi, M.; Gorji, N. Pistacia genus as a potential source of neuroprotective natural products. *Planta Med.* **2019**, 85, 1326-1350.
9. Boutemine, I.-M.; Amri, M.; Amir, Z.-C.; Fitting, C.; Mecherara-Idjeri, S.; Layaida, K.; Sennoun, N.; Berkane, S.; Cavaillon, J.-M.; Touil-Boukoffa, C. Gastro-protective, therapeutic and anti-inflammatory activities of *Pistacia lentiscus* L. fatty oil against ethanol-induced gastric ulcers in rats. *J. Ethnopharmacol.* **2018**, 224, 273-282.
10. Saljoughian, S.; Roohinejad, S.; Bekhit, A.E.-D.A.; Greiner, R.; Omidizadeh, A.; Nikmaram, N.; Mousavi Khaneghah, A. The effects of food essential oils on cardiovascular diseases: A review. *Crit. Rev. Food Sci. Nutr.* **2018**, 58, 1688-1705.
11. Magkouta, S.; Stathopoulos, G.T.; Psallidas, I.; Papapetropoulos, A.; Kolisis, F.N.; Roussos, C.; Loutrari, H. Protective effects of mastic oil from *Pistacia lentiscus* variation chia against experimental growth of lewis lung carcinoma. *Nutr. Cancer.* **2009**, 61, 640-648.
12. Catalani, S.; Palma, F.; Battistelli, S.; Benedetti, S. Oxidative stress and apoptosis induction in human thyroid carcinoma cells exposed to the essential oil from *Pistacia lentiscus* aerial parts. *PloS ONE* **2017**, 12, e0172138.
13. Dimas, K.; Hatziantoniou, S.; Wyche, J.H.; Pantazis, P. A mastic gum extract induces suppression of growth of human colorectal tumor xenografts in immunodeficient mice. *in vivo*. **2009**, 23, 63-68.

14. Aleksic, V.; Knezevic, P. Antimicrobial and antioxidative activity of extracts and essential oils of *Myrtus communis* L. *Microbiol. Res.* **2014**, 169, 240-254.
15. López-Bascón, M.; De Castro, M.L. *Soxhlet Extraction*, Elsevier; **2020**; p 327-354.
16. Fatiha, B.; Didier, H.; Naima, G.; Khodir, M.; Martin, K.; Léocadie, K.; Caroline, S.; Mohamed, C.; Pierre, D. Phenolic composition, in vitro antioxidant effects and tyrosinase inhibitory activity of three Algerian Mentha species: *M. spicata* (L.), *M. pulegium* (L.) and *M. rotundifolia* (L.) Huds (Lamiaceae). *Ind. Crops Prod.* **2015**, 74, 722-730.
17. Fatiha, B.; Didier, H.; Naima, G.; Khodir, M.; Martin, K.; Léocadie, K.; Caroline, S.; Mohamed, C.; Pierre, D. Phenolic composition, in vitro antioxidant effects and tyrosinase inhibitory activity of three Algerian Mentha species: *M. spicata* (L.), *M. pulegium* (L.) and *M. rotundifolia* (L.) Huds (Lamiaceae). *Ind Crops Prod*. **2015**, 74, 722-730.
18. Julkunen-Tiitto, R. Phenolic constituents in the leaves of northern willows: Methods for the analysis of certain phenolics. *J. Agric. Food Chem.* **1985**, 33, 213-217.
19. Mosmann, T. Rapid colorimetric assay for cellular growth and survival: Application to proliferation and cytotoxicity assays. *J. Immunol. Methods* **1983**, 65, 55-63.
20. Tabbene, O.; Karkouch, I.; Elkahoui, S.; Cosette, P.; Mangoni, M.-L.; Jouenne, T.; Limam, F. A new antibacterial and antioxidant S07-2 compound produced by *Bacillus subtilis* B38. *FEMS Microbiol. Lett*. **2010**, 303, 176-182.
21. Tabbene, O.; Karkouch, I., Elkahoui, S.; Cosette, P.; Mangoni, M.-L.; Jouenne, T.; Limam, F. A new antibacterial and antioxidant S07-2 compound produced by *Bacillus subtilis* B38. *FEMS Microbiol. Lett.* **2010**, 303, 176-182.
22. Draper, H.H.; Hadley, M. Malondialdehyde determination as index of lipid peroxidation. *Methods Enzymol.* **1990**, 186, 421-431.
23. Ellman, G.L. Tissue sulfhydryl groups. *Arch. Biochem. Biophys.* **1959**, 82, 70-77.
24. Kakkar, P.; Das, B.; Viswanathan, P. A modified spectrophotometric assay of superoxide dismutase. *IJBB*. **1984**, 21, 130-132.
25. Bampouli, A.; Kyriakopoulou, K.; Papaefstathiou, G.; Louli, V.; Aligiannis, N.; Magoulas, K.; Krokida, M. Evaluation of total antioxidant potential of *Pistacia lentiscus* var. chia leaves extracts using UHPLC–HRMS. *J. Food Eng.* **2015**, 167, 25-31.
26. Azib, L.; Debbache-Benaida, N.; Da Costa, G.; Atmani-Kilani, D.; Saidene, N.; Ayouni, K.; Richard, T.; Atmani, D. *Pistacia lentiscus* L. leaves extract and its major phenolic compounds reverse aluminium-induced neurotoxicity in mice. *Ind. Crops Prod.* **2019**, 137, 576-584.
27. Mehenni, C.; Atmani-Kilani, D.; Dumarçay, S.; Perrin, D.; Gérardin, P.; Atmani, D. Hepatoprotective and antidiabetic effects of *Pistacia lentiscus* leaf and fruit extracts. *JFDA*. **2016**, 24, 653-669.
28. Balan, K.; Prince, J.; Han, Z.; Dimas, K.; Cladaras, M.; Wyche, J.; Sitaras, N.; Pantazis, P. Antiproliferative activity and induction of apoptosis in human colon cancer cells treated in vitro with constituents of a product derived from *Pistacia lentiscus* L. var. chia. *Phytomedicine*. **2007**, 14, 263-272.
29. Gkogka, E.; Hazeleger, W.; Posthumus, M.; Beumer, R. The antimicrobial activity of the essential oil of *Pistacia lentiscus* var. Chia. *J. Essent. Oil Bear. Plant* **2013**, 16, 714-729.
30. Djebari, S.; Wrona, M.; Boudria, A.; Salafranca, J.; Nerin, C.; Bedjaoui, K.; Madani, K. Study of bioactive volatile compounds from different parts of *Pistacia lentiscus* L. extracts and their antioxidant and antibacterial activities for new active packaging application. *Food Control*. **2021**, 120, 107514.
31. Tassou, C.C.; Nychas, G. Antimicrobial activity of the essential oil of mastic gum (*Pistacia lentiscus* var. chia) on Gram positive and Gram negative bacteria in broth and in model food system. *Int. Biodeterior. Biodegrad.* **1995**, 36, 411-420.
32. Zhonglin L.; Lin, H.; Junwen, Z.; Liangzhu, C.; Zhikun, P.; Binghu, F. Synthesis and antimicrobial activity of the hybrid molecules between amoxicillin and derivatives of benzoic acid. *Drug Dev. Res.* **2021**, 82, 198-206.

33. Remila, S.; Atmani-Kilani, D.; Delemasure, S.; Connat, J.-L.; Azib, L.; Richard, T.; Atmani, D. Antioxidant, cytoprotective, anti-inflammatory and anticancer activities of *Pistacia lentiscus* (Anacardiaceae) leaf and fruit extracts. *Eur. J. Integr. Med.* **2015**, 7, 274-286.
34. Beghlal, D.; El Bairi, K.; Marmouzi, I.; Haddar, L.; Mohamed, B. Phytochemical, organoleptic and ferric reducing properties of essential oil and ethanolic extract from *Pistacia lentiscus* (L.). *Asian Pac. J. Trop. Dis.* **2016**, 6, 305-310.
35. Barbouchi, M.; Elamrani, K.; El Idrissi, M.; Chuokrad, M. A comparative study on phytochemical screening, quantification of phenolic contents and antioxidant properties of different solvent extracts from various parts of Pistacia lentiscus L. *J. King Saud Univ. Sci.* **2020**, 32, 302-306.
36. Belyagoubi-Benhammou, N.; Belyagoubi, L.; El Zerey-Belaskri, A.; Zitouni, A.; Ghembaza, N.; Benhassaini, H.; Atik-Bekkara, F.; Piras, A.; Falconieri, D.; Rosa, A. Fatty acid composition and antioxidant activity of *Pistacia lentiscus* L. fruit fatty oil from Algeria. *J. Food Meas. Charact.* **2018**, 12, 1408-1412.
37. Janakat, S.; Al-Merie, H. Evaluation of hepatoprotective effect of *Pistacia lentiscus*, *Phillyrea latifolia* and *Nicotiana glauca*. *J. Ethnopharmacol.* **2002**, 83, 135-138.
38. Klibet, F.; Boumendjel, A.; Khiari, M.; El Feki, A.; Abdennour, C.; Messarah, M. Oxidative stress-related liver dysfunction by sodium arsenite: Alleviation by *Pistacia lentiscus* oil. *Pharm. Biol.* **2016**, 54, 354-363.
39. Hamada, T.; Nowak, J.A.; Milner Jr, D.A.; Song, M.; Ogino, S. Integration of microbiology, molecular pathology, and epidemiology: A new paradigm to explore the pathogenesis of microbiome-driven neoplasms. *J. Pathol.* **2019**, 247, 615-628.
40. Ogino, S.; Nowak, J.A.; Hamada, T.; Milner Jr, D.A.; Nishihara, R. Insights into pathogenic interactions among environment, host, and tumor at the crossroads of molecular pathology and epidemiology. *Annu. Rev. Pathol.: Mech. Dis.* **2019**, 14, 83-103.
41. Ultee A.; Bennik, M.H.J.; Moezelaar, R. The phenolic hydroxyl group of carvacrol is essential for action against the food-borne pathogen *Bacillus cereus*. *Appl. Environ. Microbiol.* **2002**, 68, 1561-1568.
42. Derwich, E.; Benziane, .Z.; Boukir, A. Chemical composition of leaf essential oil of *Juniperus phoenicea* and evaluation of its antibacterial activity. *Int. J. Agric. Biol*. **2010**, 12, 199-204.
43. Chabán, M.F.; Karagianni, C.; Joray, M.B.; Toumpa, D.; Sola, C.; Crespo, M.I.; Palacios; S.M.; Athanassopoulos, C.M.; Carpinella, M.C. Antibacterial effects of extracts obtained from plants of Argentina: Bioguided isolation of compounds from the anti-infectious medicinal plant Lepechinia meyenii. *J. Ethnopharmacol.* **2019**, 239, 111930.
44. Valko, M.; Rhodes, C.; Moncol, J.; Izakovic, M.; Mazur, M. Free radicals, metals and antioxidants in oxidative stress-induced cancer. *Chem-Biol Interact*. **2006**, 160, 1-40.
45. Tiku, A.R. Antimicrobial compounds (phytoanticipins and phytoalexins) and their role in plant defense. *Co-Evolution of Secondary Metabolites*. **2020**; pp. 845-868.
46. Yisak, H.; Redi-Abshiro, M.; Chandravanshi, B.S.; Yaya, E.E. Total phenolics and antioxidant capacity of the white and brown teff [*Eragrostic tef* (Zuccagni) Trotter] varieties cultivated in different parts of Ethiopia. *Bull. Chem. Soc. Ethiop.* **2022**, 36, 749-766.
47. Hagos, M.; Chandravanshi, B.S.; Redi-Abshiro, M.; Yaya, E.E. Determination of total phenolic, total flavonoid, ascorbic acid contents and antioxidant activity of pumpkin flesh, peel and seeds. *Bull. Chem. Soc. Ethiop.* **2023**, 37, 1093-1108.
